# A neural mechanism for terminating decisions

**DOI:** 10.1016/j.neuron.2023.05.028

**Published:** 2023-06-22

**Authors:** Gabriel M. Stine, Eric M. Trautmann, Danique Jeurissen, Michael N. Shadlen

**Affiliations:** 1 Department of Neuroscience, Columbia University, New York, NY 10027, USA; 2 Zuckerman Mind Brain Behavior Institute, Columbia University, New York, NY 10027, USA; 3 McGovern Institute for Brain Research, Massachusetts Institute of Technology, Cambridge, MA 02139, USA; 4 Howard Hughes Medical Institute, Columbia University, New York, NY 10027, USA; 5 Kavli Institute for Brain Science, Columbia University, New York, NY 10027, USA; 6 Grossman Center for the Statistics of Mind, Columbia University, New York, NY 10027, USA; 7 Lead contact

## Abstract

The brain makes decisions by accumulating evidence until there is enough to stop and choose. Neural mechanisms of evidence accumulation are established in association cortex, but the site and mechanism of termination are unknown. Here, we show that the superior colliculus (SC) plays a causal role in terminating decisions, and we provide evidence for a mechanism by which this occurs. We recorded simultaneously from neurons in the lateral intraparietal area (LIP) and SC while monkeys made perceptual decisions. Despite similar trial-averaged activity, we found distinct single-trial dynamics in the two areas: LIP displayed drift-diffusion dynamics and SC displayed bursting dynamics. We hypothesized that the bursts manifest a threshold mechanism applied to signals represented in LIP to terminate the decision. Consistent with this hypothesis, SC inactivation produced behavioral effects diagnostic of an impaired threshold sensor and prolonged the buildup of activity in LIP. The results reveal the transformation from deliberation to commitment.

## In brief

LIP and SC are interconnected areas important for perceptual decision making. Stine et al. show that these areas play distinct roles during decisions. LIP represents the accumulation of evidence, whereas SC is critical for terminating this process and enacting the relevant motor plan.

## INTRODUCTION

Decisions are elemental to almost all behaviors. Innate behaviors, such as escaping a predator, and our most complex behaviors, such as choosing a career path, involve similar processes—an evaluation of evidence for a set of options and a subsequent commitment to a proposition or plan of action. Much progress has been made in understanding how the brain evaluates or accumulates evidence during the formation of decisions. Less is known about how this process is terminated and how its outcome is transformed into a relevant plan or action.

For difficult decisions informed by noisy evidence, it is often useful to accumulate many samples of evidence until some stopping criterion is achieved. Such processes comprise a class of bounded random walks and drift diffusion models.^1–4^ In monkeys trained to communicate their decisions with an eye movement, neurons in the lateral intraparietal area (LIP) represent the accumulation of evidence as the decision is being formed.^5,6^ In addition, these neurons reach a stereotyped firing rate just before the decision is reported, which has led to the hypothesis that downstream areas—particularly those involved in generating the eye movement—apply a threshold to LIP activity and terminate the decision process when that threshold is exceeded.^6–8^

A primary downstream target of LIP is the superior colliculus (SC), a conserved midbrain structure involved in orienting behaviors.^9^ In primates, SC plays a prominent role in the generation of eye movements via its descending projections to brain-stem oculomotor nuclei.^10^ SC also sends ascending projections, via the thalamus, to the basal ganglia and cerebral cortex, including LIP.^11^ Lo and Wang^8^ proposed on theoretical grounds that SC is well positioned to implement the decision threshold when decisions are communicated with an eye movement. However, experimental evidence for this proposal is lacking, and previous studies have shown that SC activity is qualitatively similar to LIP activity,^12–14^ supporting the idea that both SC and LIP represent the accumulation of evidence. The hypothesis that SC applies a decision threshold to LIP activity has not been tested directly, primarily because it is challenging to record simultaneously from neurons in LIP and SC with the same spatial selectivity. The advent of a new generation of high-density multi-channel electrodes now renders such experiments possible.

Here, we provide evidence that SC implements the decision threshold. We recorded simultaneously from populations of neurons in LIP and SC that share the same spatial preference while monkeys performed a reaction time motion-discrimination task. Single-trial dynamics in LIP approximate a stochastic drift-diffusion signal, consistent with the accumulation of noisy evidence. In contrast, single-trial dynamics in SC display bursts of activity, which terminate the decision. Simultaneous recordings suggest that these bursts reflect the implementation of a threshold applied to the drift-diffusion signal represented in LIP. We show that focal inactivation of SC impairs the threshold mechanism, leading to slower, biased decisions and prolonging the accumulation of evidence in LIP.

## RESULTS

**Figure 1.**
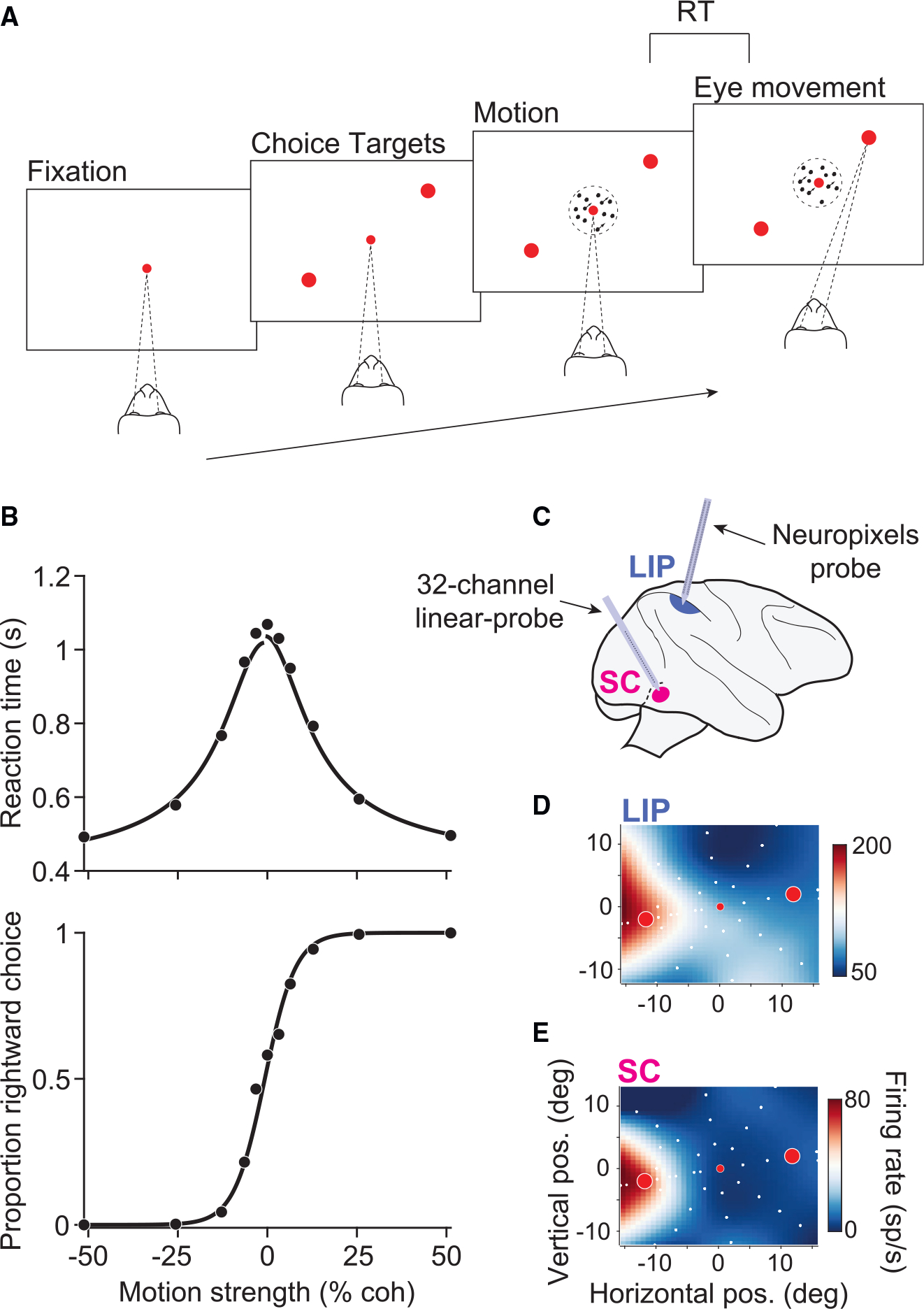
Behavioral task and experimental setup (A) RT motion discrimination task. Upon fixation and onset of the choice targets, a random-dot-motion stimulus appeared in the center of the display. Two monkeys discriminated the direction of motion and, when ready, indicated their choice with a saccade to one of two choice targets. (B) Behavioral data pooled across all sessions and both monkeys (19,749 trials). RT (top) and the proportion of rightward choices (bottom) are plotted as a function of motion strength. Positive and negative motion strengths indicate rightward and leftward motion, respectively. Solid curves depict fits of a bounded evidence-accumulation model. (C) Simultaneous recordings in SC and LIP. Populations of neurons were recorded in SC with a multi-channel V-probe and in LIP with a prototype macaque-Neuropixels probe. (D) The RF of an example LIP neuron. The color map depicts the mean firing rate, interpolated across target locations (white circles), during the delay epoch of a visually guided saccade task. Red circles depict the location of the choice targets in the RDM task in this session. (E) The RF of an example neuron in SC, recorded simultaneously as the example LIP neuron in (D).

Two rhesus monkeys performed a reaction-time (free response) version of a commonly used motion discrimination task.^6,15^ The task requires the monkey to decide the net direction—left or right—of a small patch of dynamic random dots (Figure 1A). After stimulus onset, the monkeys were free to report their decision with an eye movement to the leftward or rightward choice target. The direction and strength of the random dot motion (RDM) on each trial was selected pseudorandomly. We used a signed motion coherence to quantify the strength and direction, where positive values indicate rightward motion.

The monkeys’ choices and reaction times (RTs) depended systematically on motion strength and direction (Figure 1B). Both monkeys made no errors on the strongest motion (C=±51.2%) and approached chance performance on the weakest stimuli (C≈0%). Mean RTs were less than a half second for the strongest motion strengths and more than a second for the weaker motion strengths. These observations are broadly consistent with predictions of bounded evidence accumulation models, in which the decision is terminated when a threshold level of accumulated evidence for leftward or rightward is exceeded. A fit of such a model to the behavioral data is shown by the black curves in Figure 1B.

The effect of 100 ms pulses of weak motion on choice and RT ($\sim \frac{1}{2}$ of trials) further supports that the animals’ decisions were based on accumulated evidence. The direction (left or right) and time of the pulse was chosen randomly, independent of the direction or strength of the stimulus (see STAR Methods). Figure S1 shows that the pulses affected choices and RTs. Moreover, pulses shown early in the trial affected choices made nearly a second later. This persistent influence suggests that the monkeys’ decisions arise through the temporal integration of motion evidence and is inconsistent with non-integration strategies.^16–18^ Together, the behavioral data suggest that the monkeys accumulated evidence over time and terminated their decisions when a threshold level of accumulated evidence was exceeded.

### Single-trial analyses reveal different dynamical processes in LIP and SC

We recorded from single neurons in LIP and SC while the monkeys performed the RDM task. We targeted the intermediate and deep layers of SC, where LIP afferents terminate,^19–21^ using 16–32 channel linear probes. Each session yielded 13–36 SC neurons, most of which had similar response field (RF) locations. During the RDM task, one choice target was placed where the overlap of RFs was maximal; the other choice target was placed in the opposite hemifield (Figure 1D). In LIP, we used a prototype Neuropixels probe optimized for use in macaques (Neuropixels 1.0-NHP45^22^), yielding 54–203 neurons per session. Of these neurons, we identified subsets of 9–34 that, by chance, had RFs that overlapped those of the simultaneously recorded SC neurons (Figures 1D and 1E). We focused our analysis on these spatially aligned neurons because previous work has shown that SC-projecting neurons in LIP share similar spatial selectivity to their target neurons in SC.^21,23^

**Figure 2.**
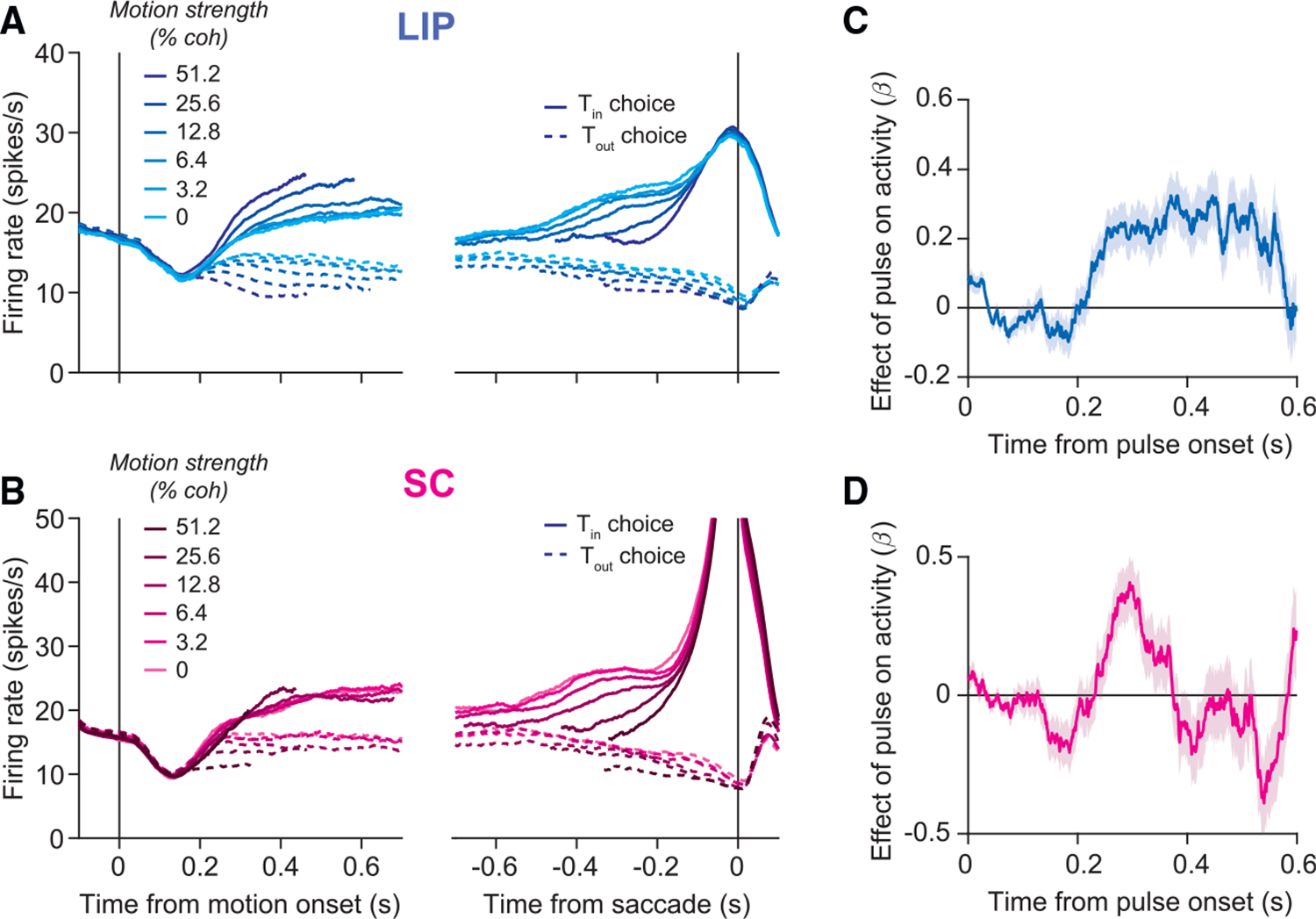
Trial-averaged responses of neurons in LIP and SC (A) Average firing rate of 164 neurons in LIP from two monkeys, aligned to motion onset (left) and saccadic onset (right). Responses are grouped by motion strength (shading) and direction (line style). Only correct trials are shown for non-zero coherences. (B) Same as in (A), but for 119 neurons in SC. (C) Effect of motion pulses on LIP activity. Consistent with temporal integration, pulses had a persistent effect on LIP activity. Shaded region represents SE. (D) Effect of motion pulses on SC activity. Pulses had a more transient effect on SC activity.

Consistent with previous results, we found that LIP and SC display similar trial-averaged activity during the RDM task (Figures 2A and 2B). Activity in both areas is modulated by the motion strength and direction, and both predict whether the trial will end in a leftward or rightward choice. The most salient difference between LIP and SC activity is the large burst of activity in SC that begins approximately 150 ms before the saccade. The areas also differ in their response to the brief motion pulses. Figures 2C and 2D display the effect of pulses on activity in LIP and SC after controlling for other factors (Equation 9). Consistent with temporal integration, pulses affect LIP activity for several hundred milliseconds (*σ* = 0.11 s, Gaussian fit). In contrast, the pulses affect SC activity more transiently (*σ* = 0.03 s; *p* < .001, likelihood ratio test), and this holds when the analysis is restricted to visuomovement prelude neurons identified by spatially selective persistent activity (Figure S2).

**Figure 3.**
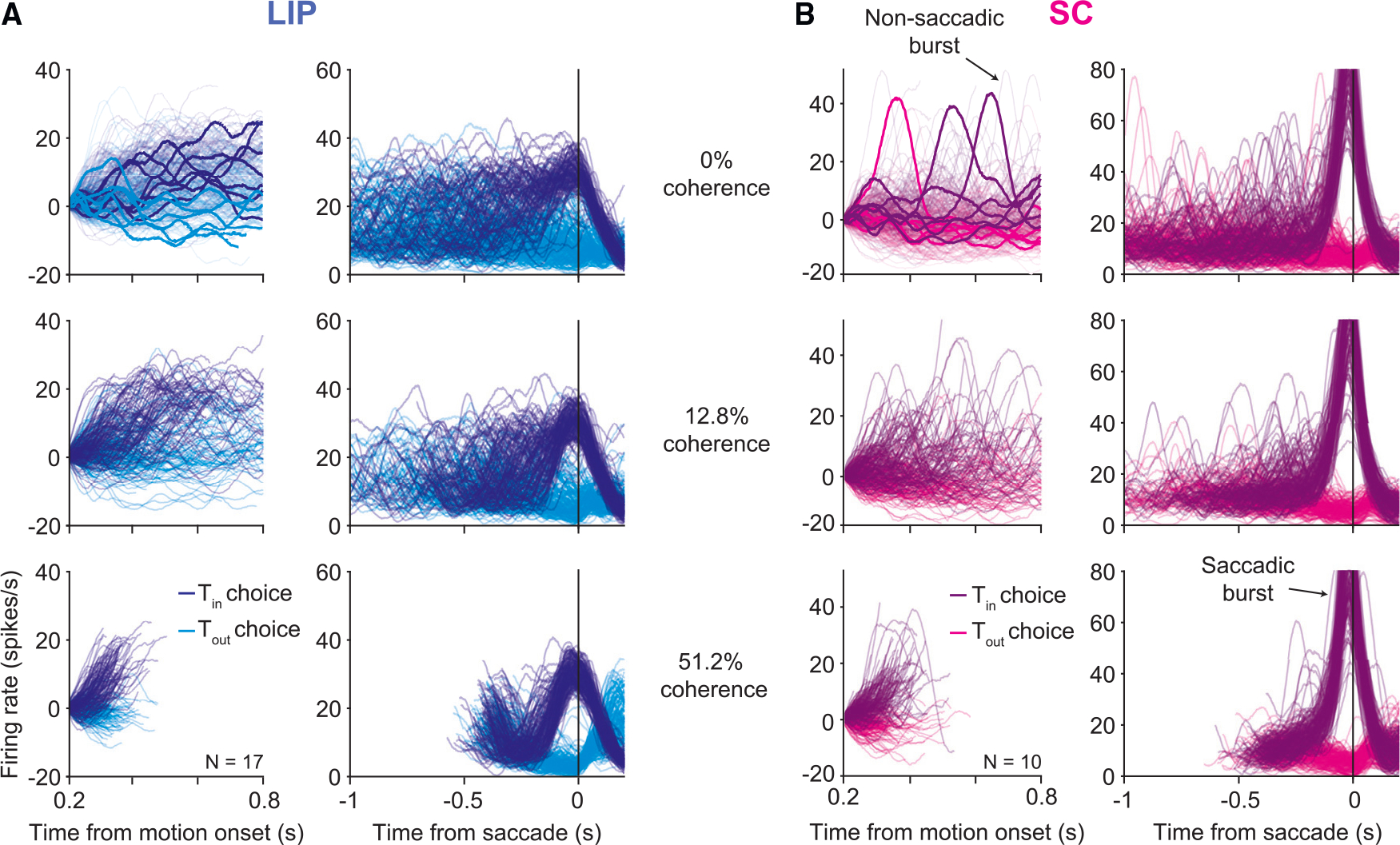
Single-trial dynamics in LIP and SC are different (A) Single-trial activity in LIP from an example session (1,797 trials). Each trace depicts the firing rate average of 17 LIP neurons on a single trial, smoothed by a Gaussian filter (*σ* = 25 ms), for three motion strengths (rows). The line color indicates the animal’s choice on that trial. Both correct and error trials are included. Left column: activity aligned to the onset of decision-related activity, ~200 ms from motion onset, until 100 ms before the saccade or 800 ms after motion onset (whichever occurs first). The rates are offset by the mean firing rate 0.18 s–0.2 s after motion onset in order to force all traces to begin at zero. In the top panel, a few representative trials are highlighted for clarity. Right column: the same trials are shown aligned to saccade initiation, without baseline offset. Note the different ordinate scales. (B) Same as in (A) but for 10 SC neurons (1,696 trials). Two types of bursts were identified in SC: saccadic bursts occurred at the end of the decision, just before the saccade, and non-saccadic bursts occurred on ~10% of trials at random times while the decision was ongoing.

The contrasting effect of motion pulses on activity in LIP and SC is consistent with the hypothesis that the two areas perform different computations. To test this further, we exploited the fact that many neurons in each area were recorded simultaneously. These recordings allowed us to compare single-trial dynamics in each area. Figure 3 shows single-trial activity from example sessions in LIP and SC, aligned to motion onset and to the saccade. Each trace represents the average of the LIP or SC population on a single trial. The upper plots depict trials from 0% coherence trials, the middle plots depict trials from an intermediate motion strength (12.8% coherence), and the lower plots depict trials from the strongest motion strength (51.2% coherence).

Single-trial responses in LIP (Figure 3A) approximate drift-diffusion—the accumulation of noise plus signal. The ramp-like trajectories in the trial-averaged data (Figure 2) reflect the accumulation of the signal (deterministic drift), whereas the accumulation of noise (diffusion) is suppressed by averaging. It is only evident in the single-trial averages. In Steinemann et al.,^24^ we show that such single-trial firing rates establish the decision variable that determines the choice and RT on each decision.

Single-trial activity in SC is qualitatively different. In addition to the large saccadic burst, single trials elucidate additional bursts of activity as the decision is being formed. These bursts resemble the saccadic bursts, but they are not associated with a saccade and exhibit smaller amplitudes (Figure S3). They are not apparent in the trial-averaged data because they are not aligned temporally to any trial event. We developed an algorithm that classifies a high-firing-rate event as a burst if its derivative exceeds a positive threshold (see STAR Methods). We found that non-saccadic bursts occurred in SC on 10.4% of trials. Crucially, they cannot be explained by the occurrence of small eye movements such as microsaccades (Figure S3). Based on the distinct dynamics in the two areas, we hypothesized that SC applies a threshold to the drift-diffusion signal in LIP, manifesting as a burst when exceeded. Typically, the burst terminates the decision, but it sometimes fails to do so.

### Evidence for a threshold computation in the superior colliculus

**Figure 4.**
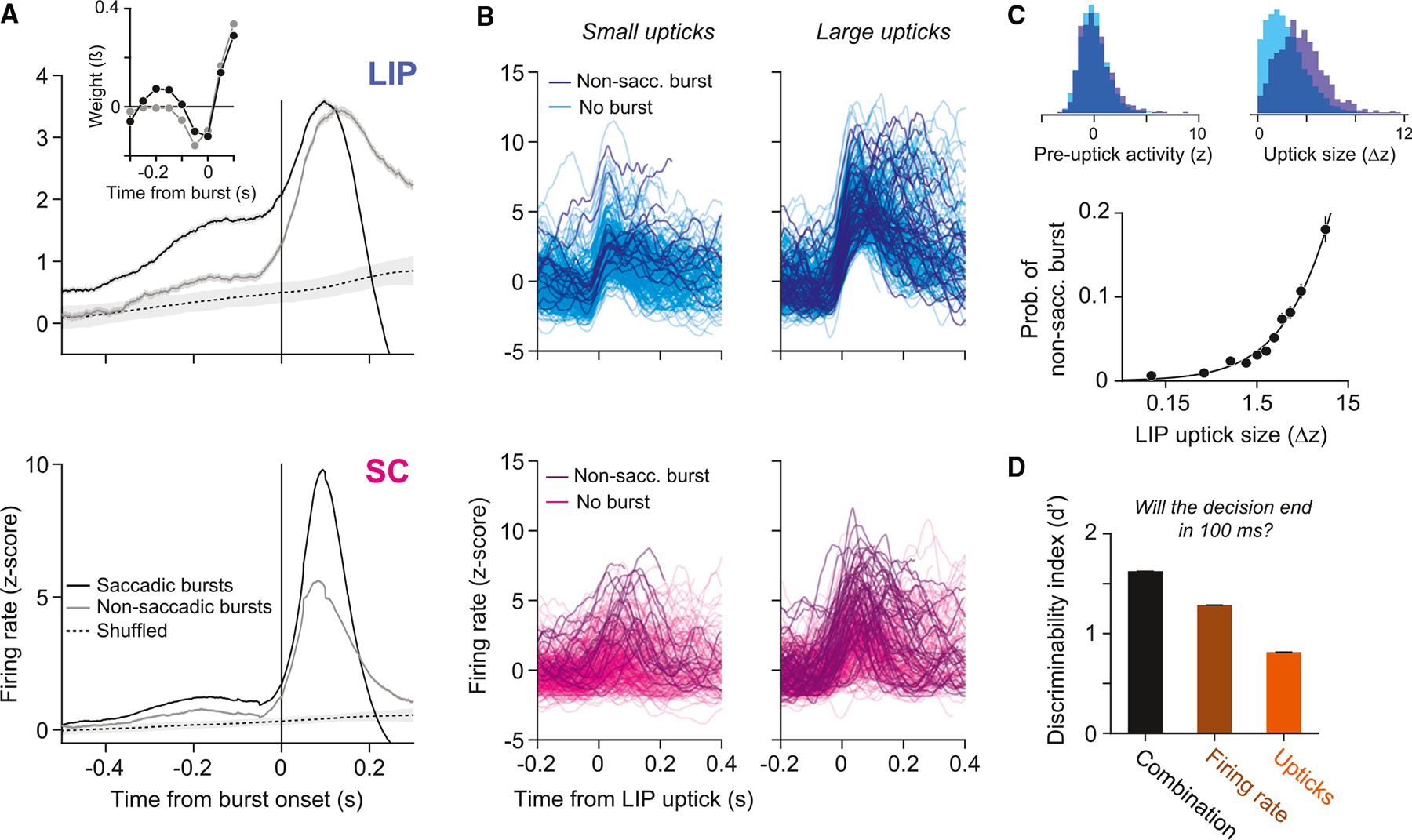
Upticks in LIP are associated with bursts in SC and decision termination (A) Average normalized activity in LIP (top) and SC (bottom) aligned to the onset of bursts in SC or to random time points that preserve the temporal statistics of the non-saccadic bursts (shuffled, dashed line; see STAR Methods). Shaded region of the dashed curve represents 95% confidence intervals (CIs). The two graphs shown in the inset are GLM-derived kernel weighting functions that best predict the saccadic and non-saccadic bursts (see STAR Methods). Time is relative to the SC event (vertical line at t = 0 in the SC graph). Both shapes indicate influence of LIP from *t≈* − 0.15 s, consistent with a positive signed derivative, or uptick. The saccadic burst is also predicted by the magnitude of the firing rate before the uptick. (B) Single-trial firing rates aligned to small (left) and large (right) upticks in LIP activity. Darker traces represent trials in which a non-saccadic burst occurred in SC within ± 50 ms of the LIP uptick. (C) Probability of a non-saccadic burst in SC increases as a function of the magnitude of the LIP uptick. Error bars represent SE. Top left shows the distribution of baseline firing rates preceding an uptick, split by whether an uptick was associated with a non-saccadic burst in SC (indigo) or not (cyan); top right shows the distribution of uptick magnitudes, split the same way. (D) Discriminability index (d′) for three quantities that use the firing rate (brown), its derivative (orange), or a combination of the two (black) to predict whether the decision will terminate in 50–150 ms. Error bars (barely visible) represent SE.

Simultaneous recordings reveal a relationship between single-trial activity in LIP and bursting in SC. Figure 4A shows the average LIP activity aligned to the onset of saccadic and non-saccadic bursts. Around the onset of the saccadic burst, LIP activity increases sharply and reaches a peak at the onset of the saccade. This uptick in activity immediately before a saccade is a known feature of LIP neurons; it is also evident in the saccade-aligned activity in Figures 2 and 3. Upticks are sometimes referred to as perisaccadic bursts in previous studies (e.g., Paré and Wurtz^23^). To avoid confusion, we will refer to “upticks” in LIP and to “bursts” in SC.

Similar upticks in LIP activity also occur at the onset of non-saccadic bursts (Figure 4A). However, instead of dropping precipitously within 200 ms, mean activity peaks and then remains elevated, resembling the persistent effect of a positive motion pulse (Figure 2C). Additional analyses suggest that non-saccadic bursts in SC are predicted by the size of the uptick in LIP. On each trial, we identified upticks in LIP activity and quantified their magnitude. Figure 4B shows example upticks and the corresponding SC activity. The darker traces depict trials in which a non-saccadic burst occurred in SC within ± 50 ms of the LIP uptick. Non-saccadic bursts were more common when upticks in LIP were large (Figure 4C). Indeed, non-saccadic bursts occurred 18.1% of the time during the largest LIP upticks, whereas they occurred in only 2.1% of randomly chosen 100 ms windows. The observation that LIP upticks occur during both types of SC bursts suggests that the termination mechanism might be more complicated than a simple threshold applied to the LIP firing rate. Note that these analyses are correlational and therefore do not provide evidence that upticks in LIP cause bursts in SC, but we provide evidence below that normal SC function is not required for upticks in LIP to occur.

The distinction between upticks that are associated with a saccade and those that are not is that the former occur on top of an elevated firing rate (Figure 4A). The result raises the possibility that upticks in LIP activity trigger bursts in SC and that saccadic bursts are triggered specifically when the uptick stems from a high firing rate. Indeed, such events accurately predict when the decision will terminate. We attempted to predict, at each moment (t) in each trial, whether the decision would terminate in a $T_{\text{in}}$ choice 50–150 ms later. We applied a criterion to one of three quantities derived from the LIP activity: (1) the firing rate, r(t), (2) its derivative, $\dot{r}(t)$, or (3) a weighted sum of the derivative and the firing rate from 50 ms earlier, $\beta_{1}\dot{r}(t)+\beta_{2}r(t-50)$. The second quantity identifies potential upticks. The third quantity captures the proposal that the combination of an uptick stemming from a high firing rate leads to decision termination. Using signal detection theory, we compared the capacity of the three quantities to discriminate, at each moment t, whether the decision will terminate. “Hits” are defined as time points in which the quantity of interest exceeds a criterion and a $T_{\text{in}}$ saccade is initiated 50–150 ms later. “False alarms” are defined as time points in which the criterion is exceeded and the decision does not terminate in a $T_{\text{in}}$ saccade (see STAR Methods). Figure 4D shows the discriminability index (d′) associated with each quantity (see Figure S4 for receiver operating characteristic curves). The discriminability of the third quantity is greatest $\left(d_{\text{combos}}^{\prime }=1.62;d_{FR}^{\prime }=1.29;d_{der}^{\prime }=0.81\right)$, consistent with our proposal. A nested model comparison confirms that the added degree of freedom is justified (logistic regression; *p* < 0.01, likelihood ratio test).

From these observations, we conclude that upticks in LIP, bursts in SC, and the subsequent termination of the decision are closely related to one another. Up to now, the relationship has been established at the level of correlations between features of single-trial responses in the two areas. LIP and SC are reciprocally connected, and there are other brain regions, such as the frontal eye field (FEF), that are known to exhibit trial-averaged responses similar to those of LIP and SC. It thus remains a theoretical possibility that SC bursts are responsible for the LIP upticks or that both events are caused by an unobserved intermediary, such as FEF. Nonetheless, the ability of bounded drift-diffusion to explain choice and RT, the observed diffusion dynamics in LIP (see also Steinemann et al.^24^), and the observed bursting in SC lead us to hypothesize that SC implements the decision threshold associated with a $T_{\text{in}}$ choice. The simultaneous recordings suggest that it may do so by sensing the combination of two features of LIP activity: an uptick and a high firing rate.

### SC plays a causal role in terminating decisions

**Figure 5.**
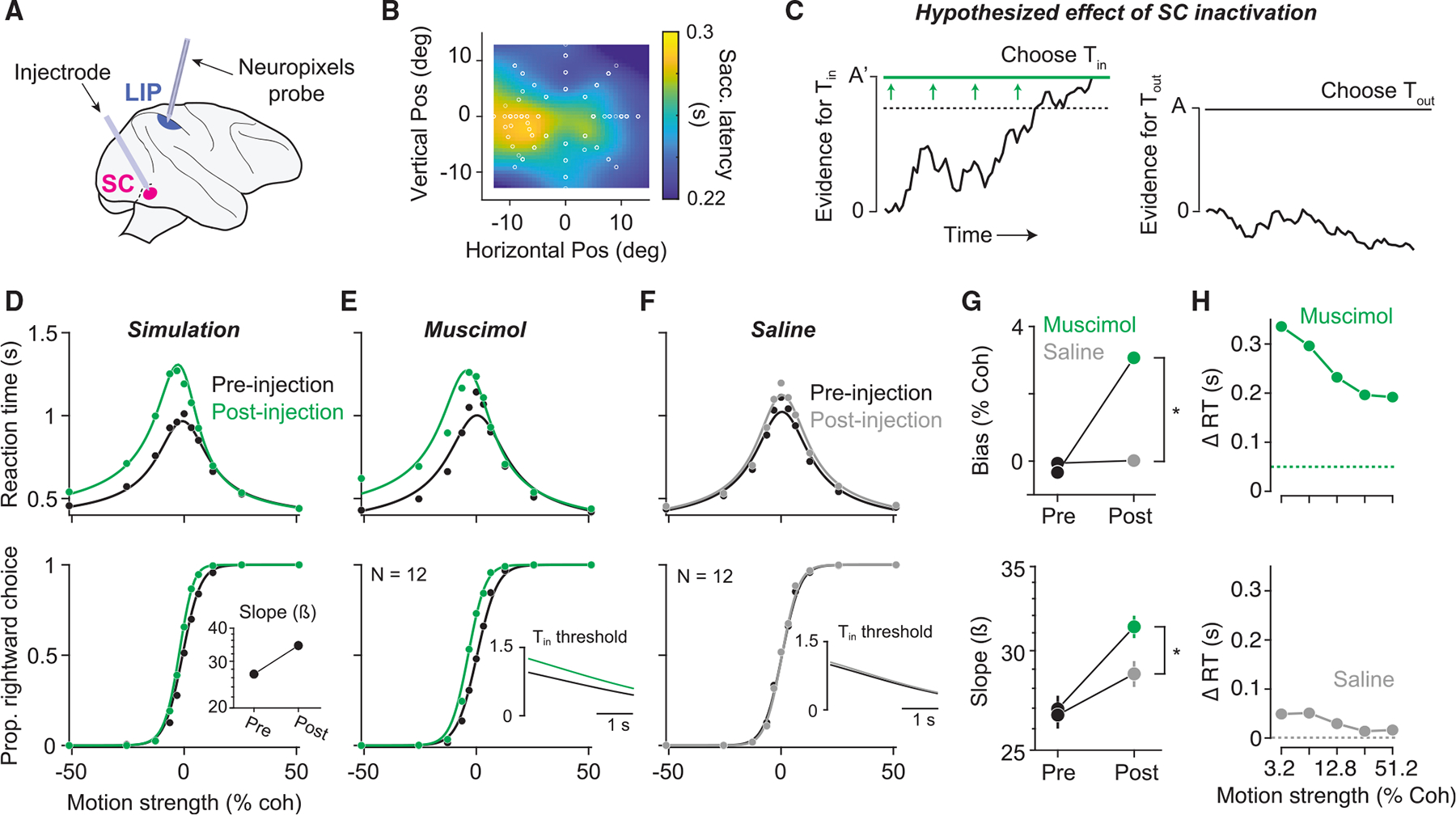
Focal inactivation of SC impairs decision termination (A) Experimental setup. Muscimol was injected unilaterally in the intermediate and deep layers of SC. On half of the experiments, Neuropixels recordings were obtained from ipsilateral area LIP. (B) Saccadic latencies measured in an instructed delayed saccade task were slowed by ~50 ms relative to pre-injection in a region of the contralateral visual field (heatmap). The left-choice target was placed in the center of this region. (C) An impairment in the mechanism for detecting the threshold might result in a requirement for stronger signals to achieve termination for $T_{\text{in}}$ choices. This is equivalent to application of higher decision threshold for $T_{\text{in}}$ choices (left panel) while leaving the threshold for $T_{\text{out}}$ choices unchanged (right panel). (D) Simulated choice proportions (bottom) and RT (top), generated by the model in (C), before (black) and after (green) a 70% increase in the $T_{\text{in}}$ decision threshold. Curves show the fit of a bounded evidence-accumulation model. Inset depicts the predicted effect of SC inactivation on the slope of the choice function. (E) Choice-RT data before (black) and after (green) unilateral SC inactivation. Inset depicts the model-derived $T_{\text{in}}$ decision threshold before and after SC inactivation. SC inactivation increased the $T_{\text{in}}$ decision threshold by 31.9%. (F) Same as (E) but for saline/sham injection experiments. (G) Effects of muscimol (green) and saline/sham (gray) injection on choice bias (top) and the slope of the choice function (bottom). Positive values for choice bias indicate a bias toward ipsilateral (rightward) choices. Asterisk denotes a statistically significant difference (*p*<.01, likelihood ratio test). (H) Muscimol-induced slowing of left-choice RTs (top) is substantially larger than the slowing of saccades in (B) (dotted line). The same analysis for the saline controls is shown on the bottom.

If the hypothesis is correct, then inactivation of SC should impair the terminating threshold. We unilaterally inactivated SC with small volumes of muscimol, a GABA agonist, while recording simultaneously from neurons in LIP with a Neuropixels probe (Figure 5A). In each session, we first characterized the effect of inactivation on visually instructed delayed saccades. Consistent with previous studies,^25–28^ focal SC inactivation increased saccadic latencies for saccades confined to a particular location in space, which we term the inactivation field (Figure 5B). During the RDM task, one choice target was placed in the center of the inactivation field ($T_{\text{in}}$); the other was placed in the opposite hemifield ($T_{\text{out}}$).

To develop an intuition for how a disrupted termination mechanism might affect decisions, it is useful to depict the decision process as a race between two processes that accumulate noisy evidence for a leftward or rightward choice, respectively (Figure 5C). In this architecture, the decision (leftward or rightward) is determined by the process that first exceeds its corresponding threshold. LIP activity in the right hemisphere, say, corresponds to the leftward accumulator. According to our hypothesis, the decision threshold resulting in a leftward choice is implemented by the SC in the right hemisphere. Inactivation of the right SC should therefore interfere with the ability to commit to a $T_{\text{in}}$ (left) choice. If the mechanism of termination is impaired, such commitment might require stronger signals from LIP, such as larger upticks, higher firing rates, or both. Conceptually, any of these changes would equate to an increase in the level of accumulated evidence required to trigger a $T_{\text{in}}$ choice—that is, an increase in the $T_{\text{in}}$ decision threshold (Figure 5C). Such an increase would give rise to three observations in the behavior. First, because there is an asymmetry in the amount of evidence required for each choice, the monkeys should be biased away from $T_{\text{in}}$ choices. Second, RTs for $T_{\text{in}}$ choices should increase because it takes longer to exceed the increased $T_{\text{in}}$ threshold. Third, the slope of the choice function (i.e., sensitivity) should increase following SC inactivation. The intuition for this last prediction is that the increased decision threshold causes decisions to be based on more accumulated evidence, which leads to better performance. Figure 5D depicts these effects in simulated data, generated by a bounded evidence accumulation model in which the $T_{\text{in}}$ decision threshold was increased by 70%.

We observed all three predicted effects in the monkeys’ behavior following unilateral SC inactivation. Figure 5E displays choice and RT data before and after muscimol injection, combined across all sessions. SC inactivation caused a bias toward $T_{\text{out}}$ choices (Figure 5G, top), an increase in RTs on $T_{\text{in}}$ choices (Figure 5H, top), and an increase in the slope of the choice function (Figure 5G, bottom). All three effects were consistent across sessions and monkeys (Figure S5). Additionally, the increase in contralateral RTs is not fully accounted for by the increase in saccadic latency (SL) observed in the delayed saccade task (Figure 5H; mean ΔRT = 0.18 s; mean ΔSL = 0.05 s). There was a general trend in which the slope of the choice function increased as the session went on, and indeed, this increase can be seen in the saline sessions (Figure 5G, bottom). However, the increase in slope following muscimol injection is significantly larger than that after saline injection (p = 0.006, likelihood ratio test). While there are many potential mechanisms that might produce a choice bias and an increase in RTs, only an increase in the decision threshold parsimoniously explains all three effects.

The conclusion is also supported by a formal model comparison. We fit choice-RT data using a drift-diffusion model with collapsing and asymmetric decision thresholds. We allowed the model to fit three extra parameters to capture the effect of SC inactivation on behavior: (1) a change in the $T_{\text{in}}$ decision threshold, (2) a change in the $T_{\text{out}}$ decision threshold, and (3) a shift in the drift-rate offset (see STAR Methods for details). Fits of this model are shown by the solid curves in Figures 5E and 5F. The insets display the model-derived $T_{\text{in}}$ decision threshold before and after muscimol injection (E) or saline injection (F). These fits suggest that muscimol injection increased the $T_{\text{in}}$ decision threshold by 31.9%, whereas it increased 6.6% following injection of saline. We also found that the effect of SC inactivation could not be fully explained by an increase in the $T_{\text{in}}$ decision threshold. The model also identifies a significant drift-rate offset that contributes to the rightward choice bias, consistent with previous studies.^14,29,30^ This offset contributes to the horizontal displacement of the choice and RT functions along the motion strength axis. It does not explain the steeper choice function and asymmetric effects on the RTs, which are diagnostic of a higher left termination threshold. This specific constellation of effects provides the causal evidence for a higher threshold for terminating leftward decisions, hence the role of SC in terminating the decision.

### The altered decision threshold is apparent in LIP activity

**Figure 6.**
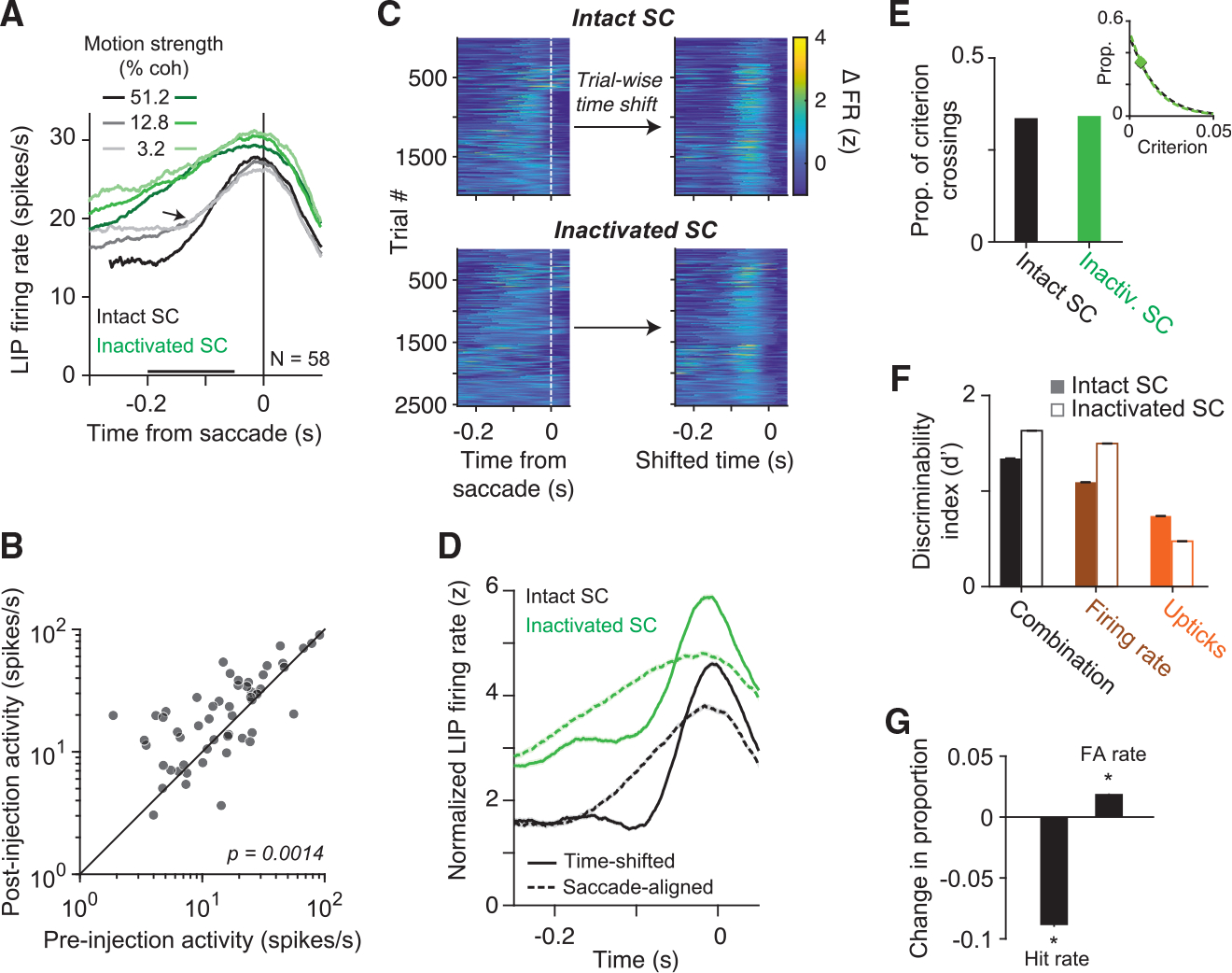
Altered readout of LIP activity following SC inactivation (A) Effect of SC inactivation on LIP population activity at the end of the decision. Mean firing rates of 58 LIP neurons before (gray curves) and after (green curves) SC inactivation are aligned to the onset of the saccade. Only $T_{\text{in}}$ choices are shown. Black bar denotes the epoch used for the analysis in (B). Arrow points to the uptick in LIP activity, which is less apparent after SC inactivation. (B) Comparison of mean firing rates of individual neurons before and after SC inactivation. Activity was significantly greater after SC inactivation (*p* = 0.001, paired t test). (C) Change in LIP firing rate on single trials relative to the saccade (left) and after trial-wise time shifting to optimize alignment of upticks (right). $T_{\text{in}}$ trials across all sessions are shown. Top, pre-inactivation trials. Bottom, after SC inactivation. Time shifting was performed independently for the two datasets (see STAR Methods). (D) Trial-averaged firing rate in LIP before (dashed curve) and after (solid curve) time-shifting. Time-shifting suggests that upticks are present in LIP activity both before and after SC inactivation, though they are not apparent in saccade-aligned, post-inactivation activity (green dashed curve, similar to (A)). (E) The proportion of time points between motion onset and saccade onset in which the firing rate derivative exceeded a criterion before (black) and after (green) SC inactivation. The criterion’s value is the same as that used in (G). Inset depicts the proportion of criterion crossings as a function of the criterion. The criterion used in the main panel is shown by the data points. The analysis suggests that SC inactivation did not reduce the frequency of upticks in LIP. (F) The same analysis as in Figure 4D applied to data before and after SC inactivation. Note the decrease in d′ associated with the derivative signal and the increase associated with the other two quantities, suggesting that upticks are less predictive of decision termination after SC inactivation. (G) Change in hit rate and false alarm rate associated with the derivative after SC inactivation, assuming no change in criterion. Asterisk denotes *p*< 0.05, Chi-squared test.

If this interpretation is correct, we might expect SC inactivation to alter the association between LIP activity and decision termination. For example, decision termination might require larger upticks, higher firing rates, or both. In half of the muscimol sessions, we recorded activity in LIP with a Neuropixels probe and identified LIP neurons with RFs that overlapped the choice target in the inactivation field. As shown in Figure 6A, inactivation of SC led to an increase in LIP firing rate in the epoch preceding decision termination (FR_pre_ = 20.6;FR_post_ = 26.4), and the increase in activity over time is more gradual. The observation is present in most neurons (Figure 6B), and it is not explained by a more general increase in firing rates at all time points or an increase in gain. Indeed, SC inactivation reduced the magnitude of visual responses to the choice targets and caused a subtle decrease in buildup rates early in the trial (Figure S6). We did not observe changes in LIP activity following saline injection (Figure S6). These analyses show that the primary effect of SC inactivation on LIP was a prolonged buildup of activity such that LIP achieved a higher firing rate at the end of the decision, which suggests that the impaired threshold mechanism acts downstream of LIP.

Consistent with this conclusion, we found that SC inactivation disrupted the relationship between upticks in LIP and decision termination. In the trial-averaged traces shown in Figure 6A, the uptick associated with the saccade before SC inactivation (indicated by the arrow) is replaced by a gradual rise in activity after SC inactivation. There are two possible explanations: (1) the uptick no longer occurs after SC inactivation because such events require SC activity, or (2) SC inactivation reduces the temporal relationship between upticks and decision termination such that the uptick disappears from the average when aligning activity to the saccade.

Several analyses support the latter explanation. The left panels of Figure 6C depict heat maps of the saccade-aligned change in firing rate on each trial, computed using a Gaussian derivative filter (see STAR Methods). Before inactivation, the upticks align consistently, across trials, with decision termination. After SC inactivation, putative upticks are visible, but the alignment appears haphazard. We used an unsupervised, linear time-shift algorithm (Williams et al.,^31^ see STAR Methods) to realign the data, which produced significantly different distributions of shift times when applied to data before or after SC inactivation (Kolmogorov-Smirnov test, *p* < 0.001). Such realignment reveals that upticks in LIP activity before SC inactivation are qualitatively similar to those after SC inactivation (Figure 6C, right; Figure 6D). Further, the realigned upticks closely resemble the burst-aligned LIP activity in Figure 4A. Application of this analysis to simulated data suggests that these observations are not an artifact of the time-shift algorithm (Figure S7; see STAR Methods). The results of this analysis demonstrate that upticks were present after SC inactivation but temporally misaligned relative to the saccade. Thus, the late upticks do not require an intact SC.

A second analysis shows that upticks throughout the trial also do not require an intact SC. We applied a criterion to the derivative of the LIP firing rate across all time points between motion onset and saccade onset and computed the proportion of upticks satisfying the criterion, before and after SC inactivation. These events occurred with almost identical frequency (Figure 6E; *P*_*pre*_ = 0.336, *P*_*post*_ = 0.342), and this held for a wide range of criteria (Figure 6E, inset). The upticks were, however, less predictive of saccades, as shown by the decrease in d′ (Figure 6F) and by the lower hit rate and higher false alarm rate in Figure 6G. The decrease in d′ was limited to upticks. The d′ associated with the firing rate and the combination of firing rate and upticks increased after SC inactivation. Together, these analyses show that SC inactivation had little to no effect on the magnitude and frequency of upticks in LIP activity. Inactivation simply dissociates upticks from decision termination.

## DISCUSSION

We provide evidence for a mechanism in the primate SC for terminating a decision based on the state of accrued evidence. It has been hypothesized that a threshold is applied to firing rates in association cortex to terminate decisions.^6–8,32–34^ How and where this thresholding occurs was unknown. In Steinemann et al.,^24^ we show that LIP represents the accumulation of noisy evidence on single decisions—the latent drift-diffusion process posited by evidence accumulation models. Here, we demonstrate that SC, which is reciprocally connected with LIP, displays distinct dynamics. These dynamics suggest a threshold mechanism, manifesting as a burst, that generates a saccade and terminates the decision process. We show that inactivation of SC impairs this threshold mechanism, leading to longer decisions and prolonged accumulation in LIP. These insights were made possible by a new generation of high-density multi-channel electrodes—45 mm Neuropixels probes^22^—that enable simultaneous recordings from many neurons with similar spatial selectivity to reveal firing rates of functionally similar neurons in the SC and LIP at the same time on a single decision.

Previous studies support the idea that SC and LIP represent similar decision-related signals.^12–14,35–40^ Based on trial-averaged activity, both SC and LIP appear to represent the accumulation of evidence (Figure 2). The averages in both areas exhibit evidence-dependent buildup (or decrease), sometimes referred to as ramping. We show that in LIP, these averages belie drift-diffusion signals on single decisions (Figure 3A; see Steinemann et al.^24^). In stark contrast to LIP, the trial-averaged spike rates in SC belie non-saccadic bursts. These bursts do not account fully for the trial averages in SC, but they are the most salient feature. It is possible that non-saccadic bursts contributed to the trial-averaged activity in previous studies of SC. For example, Cho et al.^12^ reported step-like activity in a detection task and Horwitz and Newsome^13^ found evidence for large transitions in single-trial spike trains. Our results do not preclude the possibility that small subsets of SC neurons, such as those with direction selectivity,^37^ display single-trial dynamics that match those of LIP. Given the scarcity of such neurons, resolving this question would require many more simultaneously recorded neurons. Nevertheless, our results show that neurons in SC and LIP perform different computations despite their many similarities.

Through simultaneous recordings, we provide evidence that SC applies a threshold to the drift-diffusion signal in LIP using a combination of the firing rate and its derivative. This derivative component, what we term an uptick, must accompany all positive threshold crossings, although they also occur in diffusion signals that have yet to reach a positive threshold. The association of non-saccadic bursts with LIP upticks suggests that the bursting mechanism in SC^41^ may be triggered by upticks that fail to ignite a saccadic burst unless the uptick stems from a sufficiently high baseline. Exactly why non-saccadic bursts fail to generate a saccade is unknown. However, the mechanism within SC that ignites a saccade is an active area of study.^42,43^

**Figure 7.**
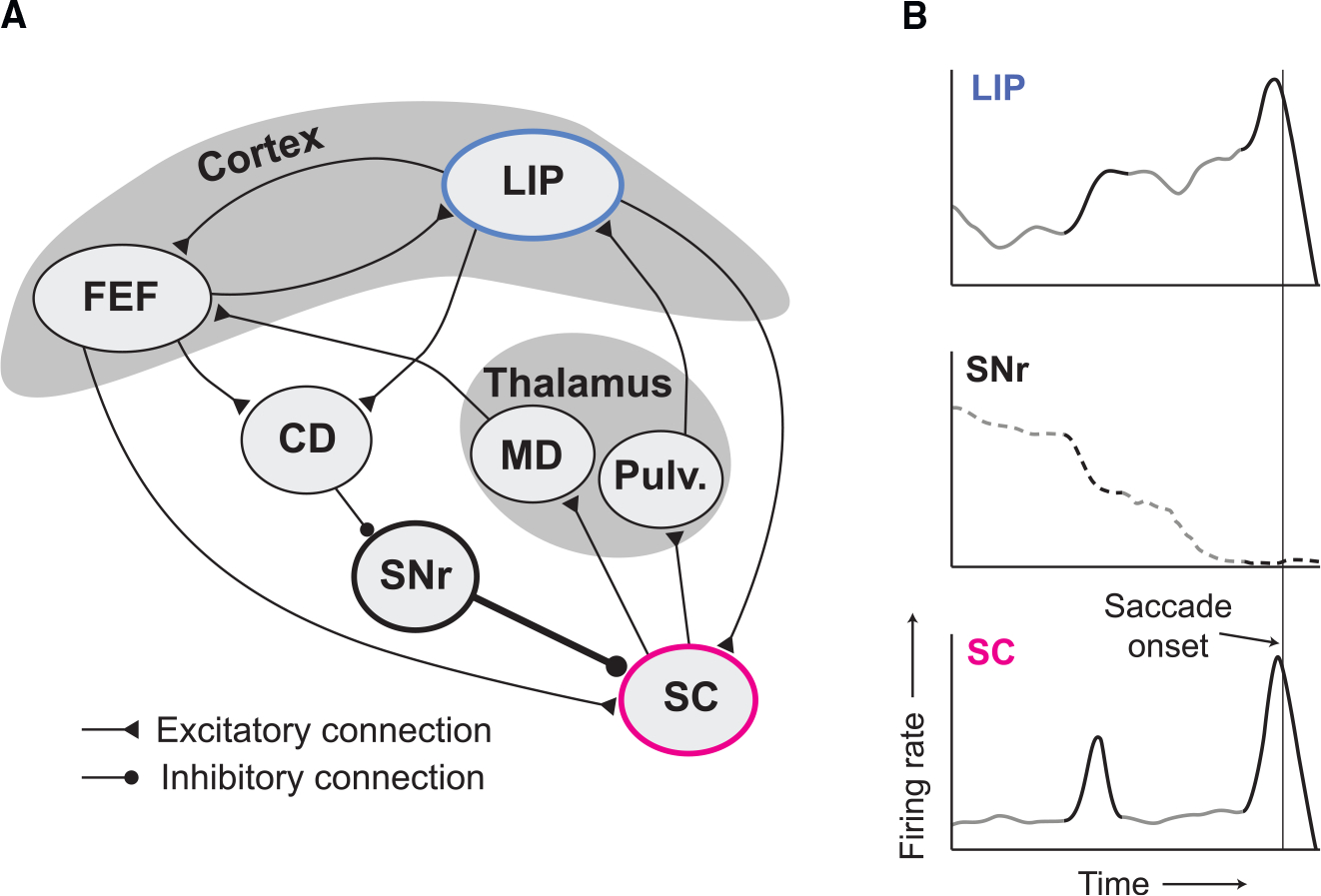
Hypothesized circuit mechanism (A) Macrocircuit. SC receives direct excitatory input from LIP and the FEF. It receives tonic inhibitory input from the SNr, an output nucleus of the basal ganglia. SC projects indirectly to the FEF and LIP via thalamic nuclei medio-dorsalis (MD) and pulvinar (Pulv.), respectively. LIP and FEF are reciprocally connected. FEF and LIP inhibit SNr by exciting neurons in the caudate nucleus that inhibit SNr. (B) A possible mechanism that determines termination events in SC. The tonic inhibitory input from SNr to SC prevents bursts caused by LIP upticks to generate a saccade. The first uptick shown in LIP (top dark segment) leads to a non-saccadic burst because SNr activity is still substantial. The second uptick is associated with suppressed activity in SNr, unleashing SC to fire a large saccadic burst. The FEF is not shown in this panel because single-trial firing rates have yet to be elucidated from this structure and the trial-averaged activity is similar to that in both SC and LIP.

The threshold mechanism we propose almost certainly involves cooperation among other visuomotor association areas, such as FEF and the basal ganglia (Figure 7). In particular, we speculate that the role of the substantia nigra pars reticulata (SNr), an output nucleus of the basal ganglia, is critical. SNr provides tonic inhibition to SC, which decreases before an eye movement.^44,45^ We envision a circuit mechanism with two properties: (1) a mechanism in SC that triggers a burst when an uptick in excitatory input is sensed, and (2) tonic inhibition of SC from SNr that is negatively correlated with the level of activity in LIP. When activity in LIP is weak, inhibition of SC is strong, such that upticks may trigger a burst in SC that is quickly suppressed—and the decision continues. When activity in LIP is strong, inhibition of SC is weak, unleashing SC to generate a large saccadic burst if it receives an uptick from cortex. This circuit mechanism is similar to the model proposed by Lo and Wang.^8^ A natural question is whether slight modifications to their model could explain our results or whether a different type of threshold mechanism would be required (e.g., Evans et al.^46^). Moreover, this mechanism predicts that perturbations of excitatory SC input should induce bursts in SC, the size of which depends on the state of SNr activity. Techniques that specifically manipulate SC afferents will likely be necessary to fully test this hypothesized circuit mechanism.

We confirmed that SC is causally involved in terminating decisions through the use of focal inactivation. We chose to inject relatively small volumes of muscimol (0.2–0.4 μL) into SC because it was essential to preserve the monkeys’ ability to perform the task. The small injection volume is known to cause only subtle changes in saccadic eye movements.^25–28^ Both monkeys were clearly capable of performing the task and made decisions that reflected the strength and direction of the RDM. However, SC inactivation caused a bias against contralateral ($T_{\text{in}}$) choices, an increase in RT on those choices, and an increase in the slope of the choice function. The pattern is diagnostic of an increase in the $T_{\text{in}}$ decision threshold. More accumulated evidence is required to commit to a $T_{\text{in}}$ choice because the termination mechanism is impaired.

The findings are not trivially explained by the effect of SC inactivation on saccadic latency. The increase in RT was coherence dependent, in accordance with the pattern prescribed by a raised termination threshold. For the lowest coherences, the increase in RT was approximately 5 times larger than the increase in saccadic latency induced by inactivation in the delayed saccade task. In addition, a short delay in the initiation of a saccade would not cause the choice bias nor the increase in slope of the choice function.

The effects of SC inactivation on decisions are distinct from those observed in studies that inactivated LIP. Unilateral inactivation of LIP led to an ipsilateral choice bias without the hallmarks of a change in termination threshold^47,48^ or no effect.^49^ In a recent study, Jun et al.^14^ found that unilateral inactivation of SC induced an ipsilateral choice bias. We uncover a potential neural correlate of this bias effect, manifesting as a slight decrease in LIP buildup rates. In contrast to our results, however, Jun et al.^14^ did not observe the hallmarks of an increased decision threshold. We speculate that their subjects may not have been terminating decisions by applying a threshold to the accumulation of evidence (see their supplemental table 4), which would explain the discrepancy in our results.

SC and LIP are reciprocally connected. It is therefore natural to wonder if SC inactivation affects behavior through an effect on LIP. However, neural recordings from LIP during SC inactivation indicate that the effects are downstream of LIP. Decision-related activity in LIP was qualitatively unaffected—just prolonged, leading to higher firing rates at the end of the decision. We interpret this as a sign of an impaired termination mechanism, which requires a stronger input. The need for a stronger input would hold whether the termination is computed by SC neurons outside the inactivation field^50^ or by a mechanism that bypasses SC altogether, for example, by exploiting the direct projection from FEF to oculomotor nuclei in the pons.^51–53^ The higher firing rates at termination are not trivially explained by the fact that decisions are prolonged. Previous studies in which animals were encouraged to slow down their decisions found either the opposite effect (in FEF; see Heitz and Schall^34^) or no change in activity at the end of the decision (LIP; see Hanks et al.^33^).

The results are likely to extend to more common settings. In our task, the oculomotor system is used to convey a decision and extract a reward. In more natural settings, it is typically used to interrogate objects and locations in the world. Such interrogation is accomplished either overtly, via an eye movement, or through covert spatial attention. The role of SC in the former has long been appreciated, but a more recent body of work has revealed its critical role in the latter.^25,26,54–59^ We view our results and SC’s role in spatial attention as two sides of the same coin. Indeed, the allocation of attention is naturally framed as a decision process informed by sensory evidence. In LIP, the accumulated sensory evidence may take the form of a priority map of objects worth inspecting,^60^ and the inspection is implemented, either covertly or overtly, when a burst occurs in SC.

Finally, distinct roles played by LIP and SC bear on a fundamental point about neural computation. LIP and SC are just two nodes in a network of brain regions that play a role in perceptual decisions reported by an eye movement. Based on average firing rates, it is tempting to conclude that the computations for forming and terminating decisions are distributed across these nodes, with no single area playing a specialized role. The present result supports a more modular organization for forming and terminating a decision. There is a certain appeal to such modularity. It allows for the possibility that some signals might affect the representation of evidence without influencing the decision threshold, or it might enable different effector systems to establish different thresholds. For example, it might be sensible to acquire more evidence to reach for something than to look at it. From an evolutionary perspective, the specific cortical-midbrain organization supports the idea that cognitive, deliberative decisions have simply co-opted primitive circuits that underlie innate perceptual decisions, such as orienting, freezing, and escape,^61^ and their study may provide the answers to more fundamental questions about how the mechanism works at a biophysical level.^8,46^ Most work has focused on how the brain forms decisions, but an understanding of how the brain commits to a decision is just as critical. Indeed, deliberation is useless or—as in the case of Buridan’s ass^62^—debilitating if it cannot be terminated.

### Study limitations

We have provided evidence for an association between features of the drift-diffusion signal in LIP (e.g., upticks) and bursting in SC. This association is correlational in nature, and we therefore do not know whether LIP activity is directly responsible for bursting in SC. Experiments that perturb LIP activity while recording in SC may be informative. The analysis of LIP activity after SC inactivation suggests that upticks in LIP do not require an intact SC. Importantly, LIP and SC are only two nodes in a highly interconnected network, and we suspect that interactions across the entire network are critical to the generation of bursts in SC (Figure 7). A second limitation concerns the generality of the finding. SC plays a pivotal role in spatial orienting movements. It remains to be seen whether SC would terminate decisions when the outcome of the decision is another type of action, although there is evidence for signals in SC that are abstracted from spatial orienting.^38,63^ It seems unlikely that SC terminates all types of decisions and all types of reports. A more plausible generalization is that termination is carried out by the circuits involved in enacting behavior consequential to the decision’s outcome, whatever the mode. This too remains to be tested.

## STAR★METHODS

Detailed methods are provided in the online version of this paper and include the following:

### RESOURCE AVAILABILITY

#### Lead contact

Further information and requests for resources should be directed to and will be fulfilled by the lead contact, Michael N. Shadlen (Shadlen@columbia.edu).

#### Materials availability

This study did not generate new unique reagents.

#### Data and code availability

All data reported in this paper have been deposited to Zenodo and are publicly available. The DOI is listed in the key resources table.

All original code has been deposited at Zenodo and is publicly available as of the date of publication. The DOI is listed in the key resources table.

Any additional information required to reanalyze the data reported in this paper is available from the lead contact upon request.

### EXPERIMENTAL MODEL AND SUBJECT DETAILS

The data in this study were obtained from two adult male rhesus monkeys (*Macaca mulatta*, 8–11 kg; Monkey M and Monkey J). All training, surgery, and experimental procedures complied with guidelines from the National Institutes of Health and were approved by the Institutional Animal Care and Use Committee at Columbia University. A head post and two recording chambers were implanted using aseptic surgical procedures and general anesthesia. Placement of the LIP chamber was guided by structural MRI. The SC chamber was placed on the mid-line and angled 38° posterior of vertical.

### METHOD DETAILS

The experiment was controlled by the Rex system^65^ on a QNX operating system. All visual stimuli were displayed on a CRT monitor (75 Hz refresh rate, 57 cm viewing distance) controlled by a Macintosh computer running Psychtoolbox.^64^ Eye position was monitored with an infrared video tracking system with a 1 kHz sampling rate (Eyelink 1000; SR Research, Ottawa, Canada).

#### Behavioral tasks

The monkeys performed two tasks in each session. In the *delayed saccade task*,^44^ a visual target appeared in the periphery as the monkey fixated a central fixation point (FP). The monkey was required to maintain fixation until the FP disappeared, thereby allowing the monkeys to execute a saccade to the target. In a memory-guided variant of the task,^66^ the target was flashed for 200 ms and the monkey was required to execute a saccade to its remembered location when the FP was extinguished. Like many intervals in our tasks, the delay period was determined by a random draw from a truncated exponential distribution:

(Equation 1) $$f\left(t\right)=\frac{\alpha }{\lambda }e^{-\frac{t-t_{min}}{\lambda }},t_{min}\leq t\leq t_{max}\;$$

where $t_{min}=0.5\;s$ and $t_{max}=1.5\;s$ define the range, λ=0.7s is the time constant, and α is chosen to ensure the total probability is unity. Below, we report the range and the exponential parameter λ. Because of truncation, the expectation $E(t)<t_{min}+\lambda$.

The RT motion discrimination task is similar to previous studies (*e.g*. Roitman and Shadlen^6^). The monkey initiated a trial by foveating the FP, after which two choice-targets appeared on the screen (one in each hemifield). After a random delay (truncated exponential: 0.25–0.7 s; λ=0.4s; Equation 1), a RDM stimulus was displayed centrally, subtending 5° of visual angle. Details of the RDM stimulus have been described previously (*e.g*. Roitman and Shadlen,^6^ and Britten et al.^67^) and its spatial and temporal statistics were identical to those used in Stine et al.^18^ The monkey was required to judge the direction (leftward or rightward) of motion embedded in the stimulus and report its decision by making a saccade to the leftward or rightward choice target. The strength of the motion (*i.e*. motion coherence) was chosen pseudorandomly on each trial from the set {0%, ± 3.2%, ± 6.4%, ± 12.8%, ± 25.6%, ± 51.2%}. After the onset of the RDM stimulus, the monkey was free to report its decision. RT is defined as the interval between the onset of the RDM stimulus and the initiation of the saccade. Correct choices were rewarded with a bolus of juice and followed by an inter-trial interval of 0.75 s. Incorrect choices were not rewarded and were followed by an additional time-out of 0–3 s; either choice on 0% coherence trials was rewarded with probability $\frac{1}{2}$. We included two incentives to discourage rushed responses: (*i*) the time-out after an error trial was a function of RT, such that faster incorrect choices were followed by longer time-outs; (*ii*) the minimum interval from motion onset to reward was 800 ms. For example, a RT of 500 ms would incur a 300 ms delay from saccade initiation to reward delivery, whereas RTs ≥ 800 ms would incur no such delay.

On approximately half of the trials, a 100 ms pulse of motion was added to the RDM stimulus. The pulse took the form of an increment or decrement of motion coherence (similar to Kiani et al^68^). For example, if the motion stimulus had a signed coherence of + 12.8%, a leftward motion pulse of strength 4.0% would change this coherence to + 8.8% for 100 ms. The strength of the pulse was calibrated to induce a weak but reliable effect on choices and RTs. The pulse strength was 4.0% coherence for Monkey M and 3.2% for Monkey J. The sign of the pulse was chosen randomly and was independent of the motion direction. The onset of the pulse was drawn randomly from a truncated exponential distribution (0.1–0.8 s, λ=0.4, Equation 1).

#### Simultaneous recordings

We recorded from well-isolated single neurons in LIP and SC using multi-channel electrodes. We targeted the ventral subdivision of LIP (LIPv; Lewis and Van Essen^69^), which is defined anatomically by its projections to the FEF and the intermediate and deep layers of SC.^19^ Identification of LIPv was guided by structural MRI and physiological criteria (presence of neurons with spatially selective, persistent activity). We targeted the deeper layers of the caudal portion of SC. These layers were identified by the transition from visual-only responses to visual, prelude, and motor responses as the recording device was driven deeper into SC. In LIP, most of the data were recorded with neuropixels probes optimized for use in macaques (Neuropixels 1.0-NHP45^22^). These probes are 45 mm in length and contain 4,416 recording sites, 384 of which are selectable to record from at one time. We conducted five recording sessions with the Neuropixels probe in Monkey M and three sessions in Monkey J, yielding 54–203 LIP neurons per session. These 8 sessions are also included in a companion paper focusing on the drift-diffusion signal in LIP.^24^ Neural data from these probes were recorded using SpikeGLX software and were synced post-hoc to behavioral data, task events, and SC activity recorded with an Omniplex system (Plexon). In Monkey M, we conducted an additional six sessions in which we used a 16-channel V-probe (Plexon) in LIP. All but one of these were not included in the dataset, because they did not yield LIP neurons with a RF that overlapped those of the SC neurons. We recorded simultaneously in the ipsilateral SC with 16-, 24-, and 32-channel V-probes (Plexon; 50–100 μm electrode spacing), yielding 13–36 neurons per session.

In each session, we first lowered the SC probe and measured the approximate RFs of the SC neurons using the delayed saccade task. Because our penetrations were approximately normal to the retinotopic map in SC, the RFs of the neurons typically overlapped with one another. We proceeded only if the center of the RFs were at least 7° eccentric, thereby ensuring minimal overlap with the RDM stimulus. Once the location of the SC RFs was confirmed, we lowered the neuropixels probe into LIP and allowed for 15–30 minutes of settling time to facilitate recording stability. The monkey then performed 100–500 trials of the delayed saccade task, with a variety of target locations, in order to precisely measure the RFs of the SC and LIP neurons. Finally, the monkey performed the RDM task until satiated–typically 1500 to 3000 trials – with a few dozen trials of the delayed saccade task included intermittently in order to measure stability of the RFs. In the RDM task, the location of the contralateral choice target was placed in the center of the SC neurons’ RFs. The other choice target was placed in the opposite hemifield, such that the two choice targets and the FP were collinear. The LIP neurons with RFs that overlapped the contralateral choice target were identified post-hoc by analyzing activity in the delayed saccade task (see below).

#### SC inactivation experiments

We unilaterally inactivated SC with small volumes of muscimol (0.2–0.4 μL, 5 μg/μL), a GABA_A_ receptor agonist. To target the intermediate and deep layers of SC, we recorded neural activity at the tip of a custom-built injectrode (30ga or 33ga cannula, similar to Chen et al.^70^) with a glass-coated platinum-tungsten microelectrode (Thomas Recording). The injection site was chosen to be at a depth that yielded strong motor activity, typically 2–2.5 mm below the surface of SC. The drug was injected with a syringe pump (Harvard Apparatus) connected to a 2 μL or 5 μL Hamilton syringe. Before lowering the injectrode, the monkey performed approximately 100 trials of the delayed saccade task and 800–1,000 trials of the RDM task to establish the pre-injection, baseline behavior. Following muscimol injection, we waited 15–30 minutes before testing for a deficit in saccade metrics. To assess the inactivation field, we measured saccadic latency and peak velocity for an array of target locations in the delayed saccade task. The presence of an effect on saccade metrics served as a positive control to ascertain whether the injection was successful; the experiment was terminated if there was not a clear increase in saccadic latency or decrease in peak velocity that was spatially confined. Following identification of the inactivation field, the monkey performed the RDM task until satiated. In the RDM task, the contralateral choice target was placed in the center of the inactivation field. In two sessions in Monkey M, the inactivation field extended foveally, which caused a large increase in fixation breaks (>10% of trials). These sessions were removed from the dataset because of the worry that an increase in fixation breaks can affect decision strategy (see Chapter 3 of Kira^71^). Saline injections followed the same protocol as that described above. In sham experiments, the injectrode was lowered into SC or just above it, but no solution was injected. We observed no difference in saline and sham control experiments, so data from the two types of controls were combined.

### QUANTIFICATION AND STATISTICAL ANALYSIS

#### Analysis of behavioral data

All analyses were performed using custom scripts in MATLAB (Mathworks). We used a bounded evidence accumulation model to fit the monkeys’ choice and RT data. The model posits that momentary evidence acquired from the stimulus is sampled sequentially and integrated until a positive or negative decision bound (± *B*) is exceeded, at which point the decision is terminated. The momentary evidence is assumed to be Gaussian with mean

(Equation 2) $$\mu =\kappa \left(C-C_{0}\right)\;$$

and unit variance per second, where κ is a constant, C is the signed motion coherence, and $C_{0}$ is a bias term (in units of signed motion strength; see Hanks et al.^72^). For the model fits in Figures 1 and S1, we used a more parsimonious version of the model that assumes flat and symmetric decision bounds. To fit this model, we maximized the combined log-likelihood of the choice data and the mean RTs, given $\kappa ,\;B,\;C_{0}$, and $t_{ND}$, where $t_{ND}$ is the non-decision time comprising sensory and motor delays that are independent of the decision process. Details of this model and the fitting process are described in Stine et al.^18^

We fit a more comprehensive variant of this model to test whether SC inactivation alters the decision bounds. In this variant, the decision bounds were allowed to be asymmetric and to collapse toward zero over time. The model is able to explain the full, choice-conditioned RT distributions, which, in principle, should provide a more precise estimate of the effect of SC inactivation because it takes into account all the data instead of just the mean RTs. We used the finite difference method^73^ to solve the Fokker-Planck equation associated with the drift-diffusion process. The probability density of the accumulated evidence (x) as a function of time (t) was derived using a time-step of 0.5 ms, and we assumed that x=0 at t=0. The predicted decision time distribution is defined as the probability density absorbed at each bound at each time-step. These distributions are convolved with a Gaussian distribution of $t_{ND}$, parameterized by $\mu_{\text{in}}$ and $\sigma_{\text{in}}$ for left choices and $\mu_{\text{out}}$ and $\sigma_{\text{out}}$ for right choices. The convolutions yield the predicted distributions of RT.

The $T_{\text{in}}$ and $T_{\text{out}}$ decision bounds before SC inactivation were logistic functions of time:

(Equation 3) $$B_{\text{in}}\left(t\right)=B_{\text{in}0}{\left(1+e^{a\left(t-d\right)}\right)}^{-1}$$

(Equation 4) $$B_{\text{out}}\left(t\right)=B_{\text{out0}}{\left(1+e^{a\left(t-d\right)}\right)}^{-1}$$

where a and d are shape parameters and a is constrained to be non-negative. They are shared between the two bounds, such that the difference between the $T_{\text{in}}$ bound and the $T_{\text{out}}$ bound is only determined by the $B_{\text{in0}}$ and $B_{\text{out0}}$ parameters. A variety of other bound shapes (*e.g*. linear, hyperbolic, and exponential collapse) produced similar results.

To avoid over-fitting, we limited the number of parameters that were allowed to differ before and after SC inactivation. These five parameters are denoted by a prime symbol to indicate the value they take after SC inactivation. The model comprised 15 parameters in total:

(Equation 5) $$\theta =\left\{\kappa ,B_{\text{in}0},B_{\text{out}0},a,d,\mu_{\text{in}},\sigma_{\text{in}},\mu_{\text{out}},\sigma_{\text{out}},C_{0},B_{\text{in}0}^{\prime },B_{\text{out}0}^{\prime },C_{0}^{\prime },\mu_{\text{in}}^{\prime },\sigma_{\text{in}}^{\prime }\right\}$$

To ensure that the model was properly constrained, we empirically estimated $\mu_{\text{in}}^{\prime }$ and $\sigma_{\text{in}}^{\prime }$ using the increase in the mean and standard deviation of saccadic latencies observed in the delayed saccade task. Recent work has shown that such constraints on the non-decision time parameters are critical to reveal cases of model mis-specification.^18^ We fit data from before and after inactivation simultaneously, using Bayesian adaptive direct search (BADS^74^) to maximize the log-likelihood of the data given the parameters. To calculate Bayesian credible intervals of the fitted model parameters, we approximated the posterior distribution of parameters using variational bayesian monte carlo (VBMC^75^).

We also summarized the effect of SC inactivation on behavior using a logistic function fit to the choice data alone. The proportion of rightward choices as a function of coherence is given by

(Equation 6) $$P_{\text{right}}={\left[1+e^{-\left(\beta_{0}+\beta_{1}C\right)}\right]}^{-1}\;$$

where $\beta_{0}$ determines the left-right bias and $\beta_{1}$ determines the slope of the choice function. To test whether the effect of muscimol injection on bias and slope was significantly different from that of saline injection, we performed a nested model comparison. The full model was defined by the following equation:

(Equation 7) $$P_{\text{right}}={\left(1+e^{-\theta }\right)}^{-1}$$

(Equation 8) $$\begin{array}{cc} \theta =\beta_{0}+\beta_{1}C+\beta_{2}I_{Inj}+\beta_{3}I_{Inj}C+\beta_{4}I_{M}+\beta_{5}I_{Inj}I_{M}+\beta_{6}I_{Inj}I_{M}C & \end{array}$$

where $I_{M}$ is an indicator variable denoting the session type (saline or muscimol) and $I_{Inj}$ is an indicator variable denoting whether the trial came before or after injection. We tested for a significant interaction between $I_{Inj}$ and $I_{M}$ (bias effect) or between $I_{Inj},\;I_{M}$, and C (slope effect) by removing these terms and comparing the resulting deviance to that of the full model with a likelihood ratio test.

#### Analysis of neural data

Single units were identified in the neural data using Kilosort 2.0 and manual curation in Phy. In LIP, we restricted our analyses to neurons with RFs that overlapped the contralateral choice target (and the SC RFs). RFs in LIP were identified post-hoc by characterizing activity in the delayed saccade task, and whether the identified RF overlapped the contralateral target was determined by visual inspection. This determination was made before analyzing activity in the RDM task. The final data set includes 1,084 LIP neurons (582 from Monkey M), of which 164 (79 from Monkey M) had RFs that overlapped the contralateral choice target.

For all analyses, spike trains were discretized into 1 ms bins and convolved with a 50 ms rectangular filter. For visualization purposes, the single-trial traces displayed in Figure 3 were produced by convolving spike trains with a Gaussian kernel (σ=25ms). The trial-averaged responses in Figures 2A and 2B were computed by averaging activity over all neurons aligned to either motion onset or saccade onset. To avoid averaging over stereotyped dynamics associated with motion onset and the saccade, we implemented an attrition rule for each event alignment and brain area. For the motion-aligned averages, we did not include spikes that occurred within 70 ms of the saccade in LIP and within 170 ms in SC. For the saccade-aligned activity, we did not include spikes that occurred within 200 ms of motion onset in both areas.

We used linear regression to analyze the effect of motion pulses on activity in each area as a function of time, relative to pulse onset. The regression includes the pulse and other factors that might affect the firing rate as a function of time on each trial:

(Equation 9) $$r(t)=\beta_{0}(t)+\beta_{1}(t)I_{p}+\beta_{2}(t)C+\beta_{3}(t)I_{\text{choice}}$$

where r(t) is the firing rate after subtracting the mean activity in the epoch from 0–100 ms from pulse onset, $I_{p}$ is a signed indicator variable signifying the direction of the pulse (left or right), and $I_{\text{choice}}$ is a signed indicator variable signifying the monkey’s choice. Figures 2C and 2D depict $\beta_{1}(t)$, which captures the effect of the pulse on activity as a function of time. The adjustment to r(t) forces $\beta_{1}$ to begin at a value of approximately zero. To quantify the persistence of the pulse effect on neural activity, we fit $\beta_{1}(t)$ of each area with a Gaussian function, 𝒩(μ,σ), with two extra parameters, which scale and shift the function on the ordinate. The persistence is defined as the standard deviation (σ) of the Gaussian. We tested for a significant difference in persistence between LIP and SC by fitting $\beta_{1}(t)$ of both areas with a single σ parameter. We then compared the resulting log-likelihood to that of a model that allowed for different σ parameters using a likelihood ratio test.

To analyze single-trial activity, we computed the average firing rate of the simultaneously recorded neurons. These averages are standardized (z scored), using the mean and standard deviation of activity from the epoch 0–400 ms after motion onset. The standardization ensures that any single session does not dominate subsequent analyses. Our conclusions do not depend on the specific choice of epoch.

We developed a simple algorithm to detect bursts of SC activity. On each trial, the algorithm first applies a threshold (3.5 σ) on the population firing rate to identify high firing-rate events. If this first threshold is exceeded at t=τ, a second threshold is applied to the smoothed discrete derivative of the firing rate in the epoch τ−80 to τ+120ms. Smoothing is accomplished by a ± 50 ms running mean, and the threshold is 250 sp/s^2^. The event is classified as a burst if both thresholds are exceeded, and we define the burst onset time as time the derivative threshold is first exceeded. We hand-tuned the two thresholds and the epoch surrounding τ by visually inspecting the performance of the algorithm on hundreds of randomly chosen trials. We used the same parameters to identify the non-saccadic bursts – those that occur more than 250 ms before saccade initiation.

The algorithm is admittedly arbitrary. Its only purpose is to regularize the identification of events that are already evident by visual inspection and assign them times. All analyses were repeated using a range of threshold values. Such variation led inevitably to small quantitative changes in the burst frequency, onset times, and magnitudes, but the conclusions we draw are robust. Notably, the algorithm only detects the rising edge of the burst; it does not require a subsequent decrease, or falling edge, in activity. Thus, the burst-like activity profile that we observed in SC was not guaranteed to be discovered by the algorithm. Indeed, we applied the same algorithm, with the same parameters, to the LIP activity and observed a qualitatively different activity profile (Figure S4). Finally, we reached similar conclusions when we used more complex algorithms that also applied a threshold on the second derivative and/or required a subsequent decrease in activity. Nevertheless, many aspects of the burst-detection algorithm are arbitrary–in particular, the time assigned to burst onset. Therefore, we do not make claims about the relative timing of events in LIP and SC based on the identified burst onset times.

We compared burst-aligned activity in LIP and SC to random alignments that preserved the temporal statistics of the burst onset times (shuffled alignment in Figure 4A). We randomly selected n trials, where n is the total number of non-saccadic bursts, and generated a “fake” burst time for each by taking a random sample from the observed distribution of non-saccadic burst times. We aligned activity to these “fake” burst times and computed the mean. We repeated this procedure 2000 times to calculate 95% confidence intervals.

To produce the kernels depicted in Figure 4A, *inset*, we used a generalized linear model to fit the probability of saccadic and non-saccadic bursts in SC using LIP activity, rendering the temporal kernel weights in Figure 4A, *inset*. We grouped the LIP population firing rate on each trial into independent, 50 ms bins. The probability of a burst in SC at time t as a function of LIP activity spanning k bins was described by

(Equation 10) $$P_{\text{burst}}={\left(1+e^{-\theta }\right)}^{-1}$$

(Equation 11) $$\theta =\beta_{0}+\sum_{k}\;\;\beta (k)r(t+k)$$

where k ranged from −0.3 s to 0.1 s. The analysis was performed separately for saccadic and non-saccadic bursts. The weights in Figure 4A, *inset* depict β(k), fitted using maximum likelihood estimation.

To identify upticks in LIP, we first computed the discrete derivative of the population firing rate as a function of time, $\dot{r}(t)$, on each trial and smoothed it with a 100 ms sliding window. For the analyses in Figures 4B and 4C, we identified a single “uptick” on each trial, which was defined as the maximum of $\dot{r}(t)$ from 200 ms after motion onset to 250 ms before saccade initiation. The uptick size is the difference in firing rate 75 ms before and after the maximum.

We used both r(t) and $\dot{r}(t)$ to predict, at each time point t, whether the decision would terminate in a $T_{\text{in}}$ choice 50–150 ms later. In the context of *signal detection theory*, the time points on each trial defined as *signal present* are $-50\leq t-t_{sac}\leq 150\;ms$, where $t_{sac}$ is the time of initiation of a $T_{\text{in}}$ saccade. All other time-points are defined as *signal absent*. The hit rate is defined as the proportion of *signal-present* time points, across all trials, in which the quantity of interest exceeded a criterion. Likewise, the false alarm rate is defined as the proportion of *signal absent* time points, across all trials, in which the quantity of interest exceeded the same criterion. The quantity of interest depended on the model. It was r(t) for the firing rate model, $\dot{r}(t)$ for the upticks model, and $\beta_{1}r(t-50)+\beta_{2}\dot{r}(t)$ for the combination model. The β terms in the combination model were optimized using logistic regression. For each model, the quantity was compared to 100 linearly spaced criterion values and the $d^{\prime }$ was calculated using the hit rate and false alarm rate yielded by the optimal criterion assuming an uninformative prior. $d^{\prime }$ was calculated as

(Equation 12) $$\left.d^{\prime }=z\text{(Hit}\;\text{rate}\right)-z\left(\text{FA}\;\text{rate}\right)$$

where z is the inverse CDF of the standard normal distribution. To calculate the standard error of $d^{\prime }$ values, we calculated $d^{\prime }$ for 2000 bootstrapped datasets and took the standard deviation of the resulting sampling distribution. To calculate the change in hit rate and false alarm rate after SC inactivation (Figure 6F), we found the optimal criterion for the data before SC inactivation, assuming an uninformative prior, and applied it to the data after SC inactivation.

We used a simple version of the time-warping algorithm (“shift only” version) developed by Williams et al.^31^ to align events in LIP activity before and after SC inactivation. The algorithm shifts time on each trial in order to maximize the alignment of reproducible patterns in the neural activity. We first applied a Gaussian derivative filter (σ=0.3s) to the LIP population firing rate on each trial in order to compute the change in firing rate over time, ΔFR(t). We then applied the algorithm to the saccade-aligned ΔFR(t) on $T_{\text{in}}$ trials using data from −0.3s to 0s before the saccade, allowing for a maximum time shift of 0.2s on each trial and using a smoothness parameter of 10 (default value). To avoid the algorithm from aligning events that occurred early in the decision, we removed the first 200 ms of each trial, which encompasses the stereotyped dip in activity following the onset of the motion stimulus, and restricted the analysis to trials with RTs > 0.4 s. Finally, we computed the mean firing rate of the realigned data. This procedure was performed separately for data collected before and after SC inactivation.

To test whether the results are an artifact of the time-shift algorithm, we applied the same analysis to surrogate pre- and post-inactivation datasets, simulated as random Gaussian processes (Figure S7). The simulated datasets match the number of trials, the mean saccade-aligned activity (as a function of time), and the autocovariance of the experimental datasets. The simulated data do not contain any inherent misalignment because the same generating parameters give rise to each simulated trial. We applied the time-shift algorithm to the simulated pre- and post-inactivation datasets and, for each, computed the difference between the peak trial-averaged activity of the realigned simulated data and that for the saccade-aligned simulated data. We computed this quantity for 200 pairs of simulated datasets. The resulting distribution represents the expected improvement in alignment under the null hypothesis that the activity is not temporally misaligned. We compared this null distribution to the difference statistic derived from the experimental data (Figure S7B).

## Supplementary Material

MMC1

## Figures and Tables

**KEY RESOURCES TABLE:** 

REAGENT or RESOURCE	SOURCE	IDENTIFIER

Deposited data		

Experimental data	This paper	https://doi.org/10.5281/zenodo.7946011

Software and algorithms		

Original code	This paper	https://doi.org/10.5281/zenodo.7946011
PsychToolbox	Brainard^64^	http://psychtoolbox.org/
MATLAB	MathWorks	https://www.mathworks.com/products/matlab.html

## Highlights

- Single-trial analyses reveal distinct dynamics in LIP and SC during decisions
- Bursting in SC is consistent with a threshold mechanism that terminates the decision
- SC inactivation impaired decision termination, causing animals to be more deliberative
- SC inactivation prolongs the accumulation of evidence in LIP
