# Supplementary material for: A neural mechanism for terminating decisions
Source: Neuron. Author manuscript; Available in PMC 2023 Oct 11. (PMC10565788; doi:10.1016/j.neuron.2023.05.028)
Supplement: MMC1 [file NIHMS1912430-supplement-MMC1.pdf]

Neuron, Volume *111*

Supplemental information

A neural mechanism for terminating decisions

Gabriel M. Stine, Eric M. Trautmann, Danique Jeurissen, and Michael N. Shadlen

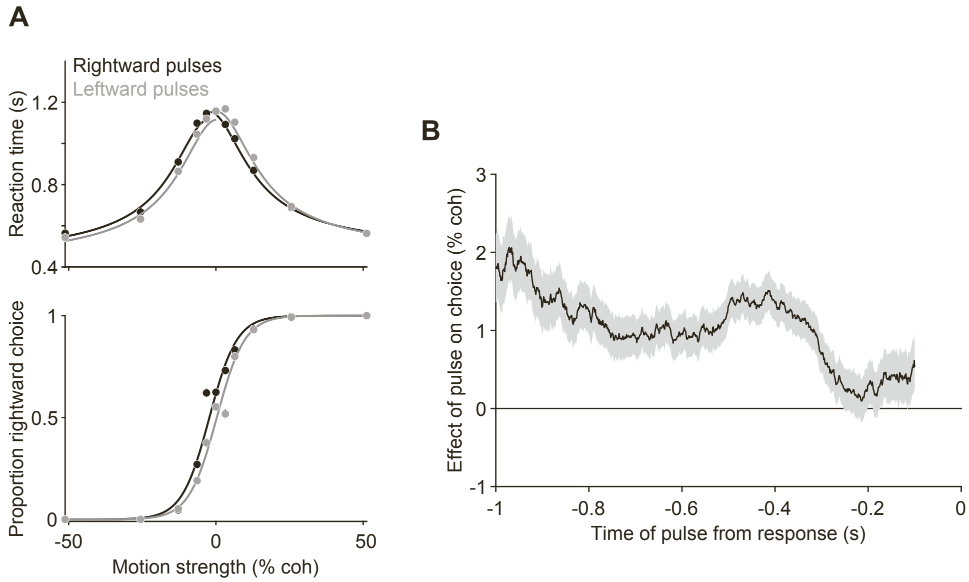

Figure S1: Effect of motion pulses on behavior, Related to Figure 1. A, Choice (*bottom*) and reaction time (*top*) data as a function of motion strength from two monkeys, plotted separately for trials with leftward (grey) and rightward (black) motion pulses. The pulses had a biasing effect equivalent to shifting the choice function left or right by ± 1.4 % coh ($p < .001$, likelihood ratio test). **B,** Effect of motion pulses on choices as a function of time from the response. Pulses had a persistent effect on choices, consistent with temporal integration of motion evidence. Shading, standard error.

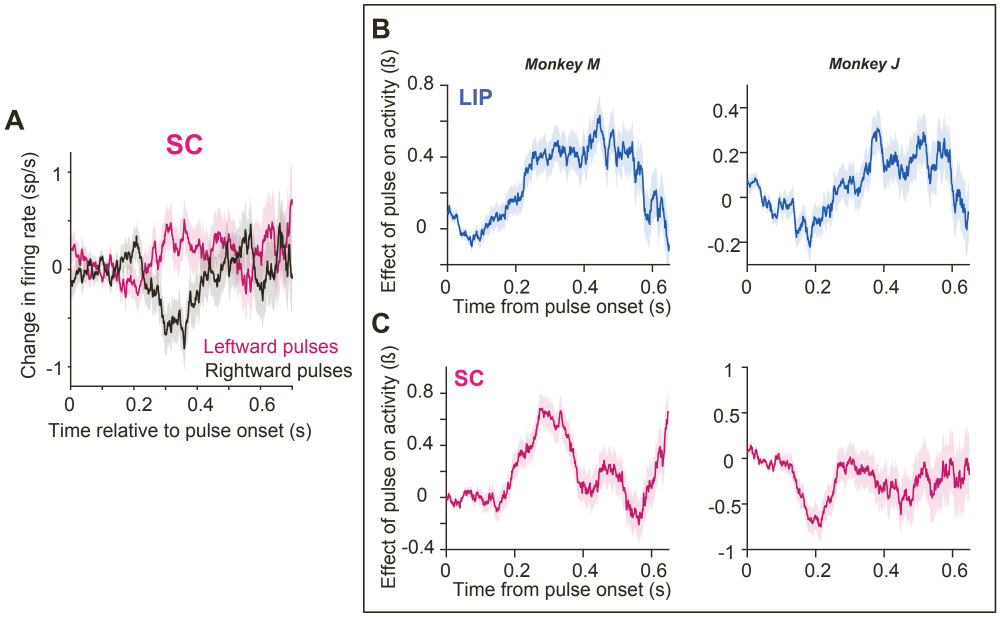

Figure S2: Additional analyses of the effect of motion pulses on LIP and SC activity, Related to Figure 2. A, Average activity, aligned to pulse onset, of 79 SC neurons identified as having spatially-selective persistent activity during a memory-guided saccade task (*i.e.*, visuomovement prelude neurons). Activity is baseline subtracted and plotted separately for leftward (magenta) and rightward (black) pulses. These neurons comprise a subset of those displayed in Fig 2. (Shading shows s.e). **B,** Effect of motion pulses on LIP activity in Monkey M (left) and Monkey J (right). **C,** Same as **B** but for SC activity.

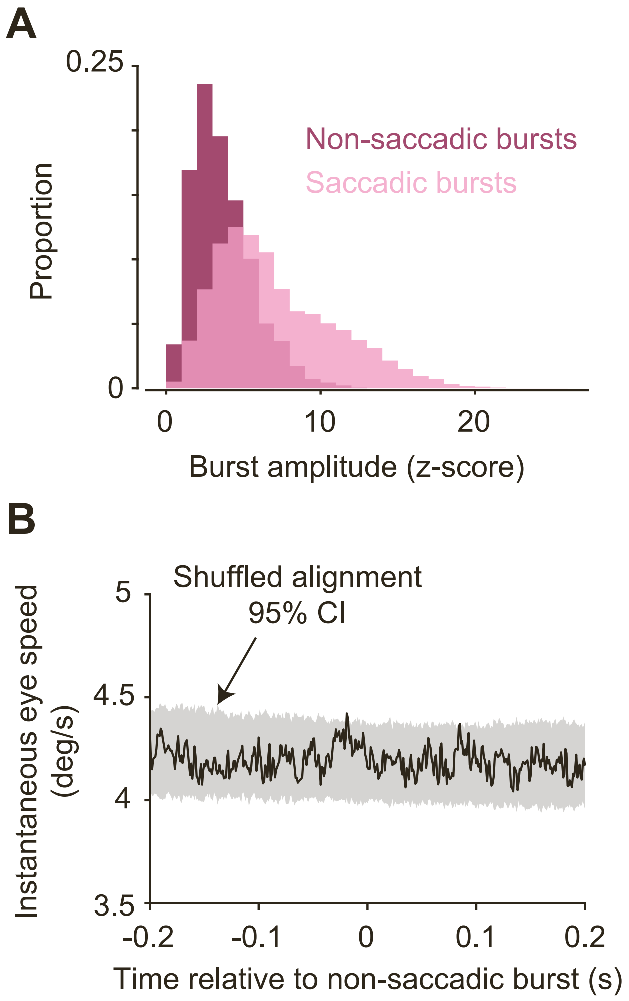

Figure S3: Additional analyses of SC bursts, Related to Figure 3. A, Distribution of burst amplitudes (peak z-score) for non-saccadic (magenta) and saccadic bursts (pink). **B,** Non-saccadic bursts are not associated with eye movements. Black trace shows the average instantaneous eye speed ($\|\vec{v}\|$) aligned to the onset of non-saccadic burst. The shaded region shows the 95% confidence interval of the average eye speed associated with 2,000 bootstrapped, random alignments. For each trial, we chose a time-point for alignment by randomly sampling the distribution of burst-onset times, computed the average eye speed aligned to these random samples, and repeated the exercise 2,000 times.

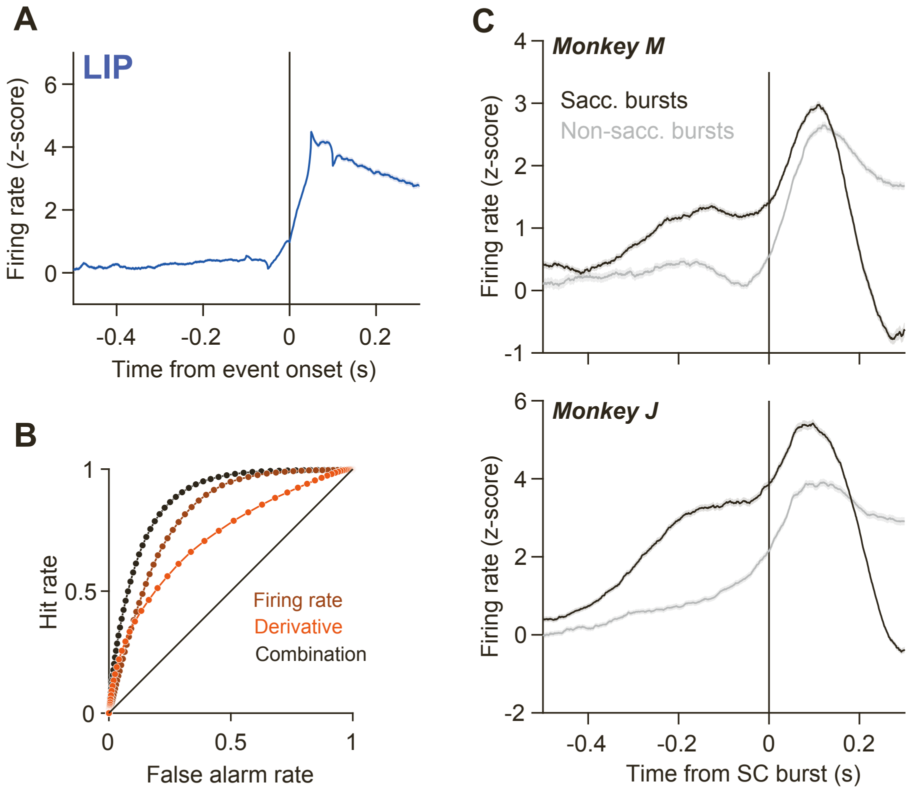

Figure S4: Lack of bursting in LIP and ROC curves associated with predictions of decision termination, Related to Figure 4. **A,** LIP activity aligned to events identified by the burst-detection algorithm. The same algorithm used to identify SC bursts in Figure 4A is applied here to LIP activity. Alignment of LIP activity to the detected events renders an uptick—a natural feature of a diffusion process upon accumulation of positive momentary evidence. It is qualitatively different from the biphasic—symmetric rise and fall—of SC bursts. **B,** Receiver operating characteristic (ROC) curves associated with the d′ values in Figure 4D. The hit rate and false alarm rate yielded by each model is plotted for 100, linearly-spaced criterion values. d′ values are computed using the optimal criterion (assuming uninformative prior and neutral value; see Methods). **C,** Average LIP activity aligned to SC bursts for Monkey M (top) and Monkey J (bottom).

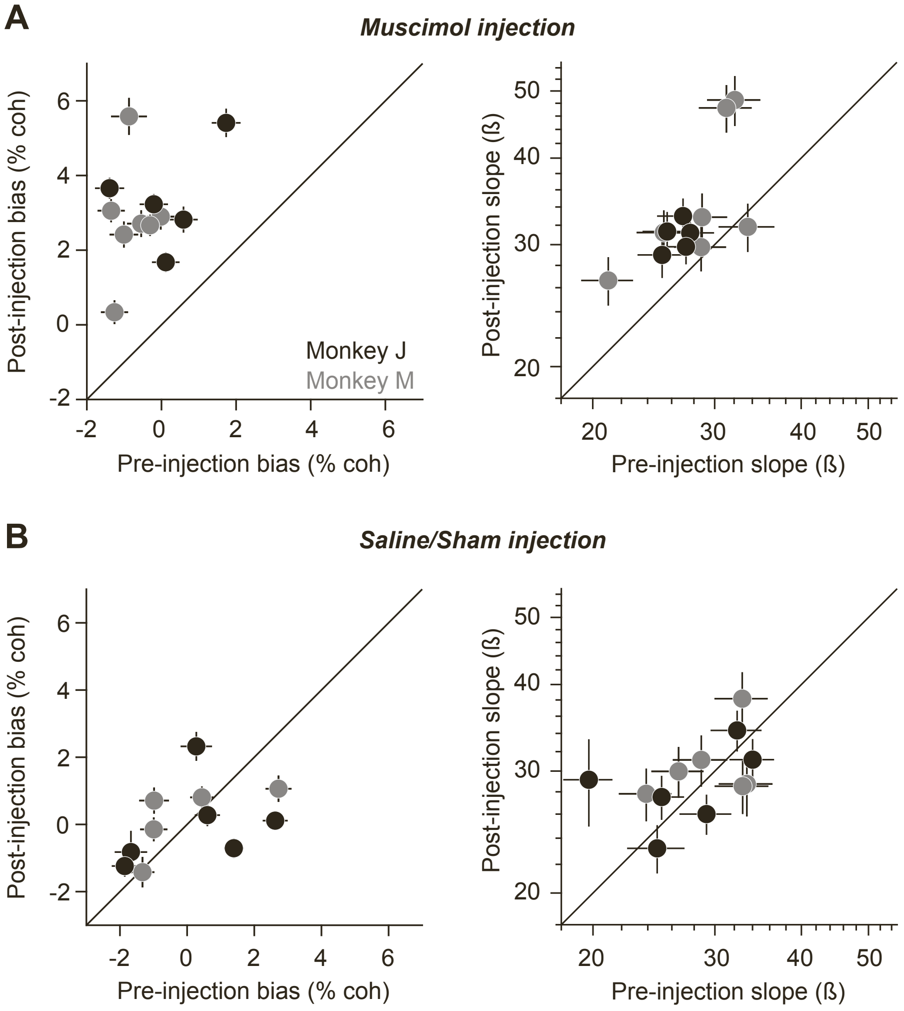

Figure S5: Effect of muscimol and saline injection on choice data for individual sessions, Related to Figure 5. **A,** Change in bias (*left*) and slope (*right*) following muscimol injection. Each data point depicts a single session. **B,** Same as **A** but for saline or sham injection sessions. Error bars represent standard error.

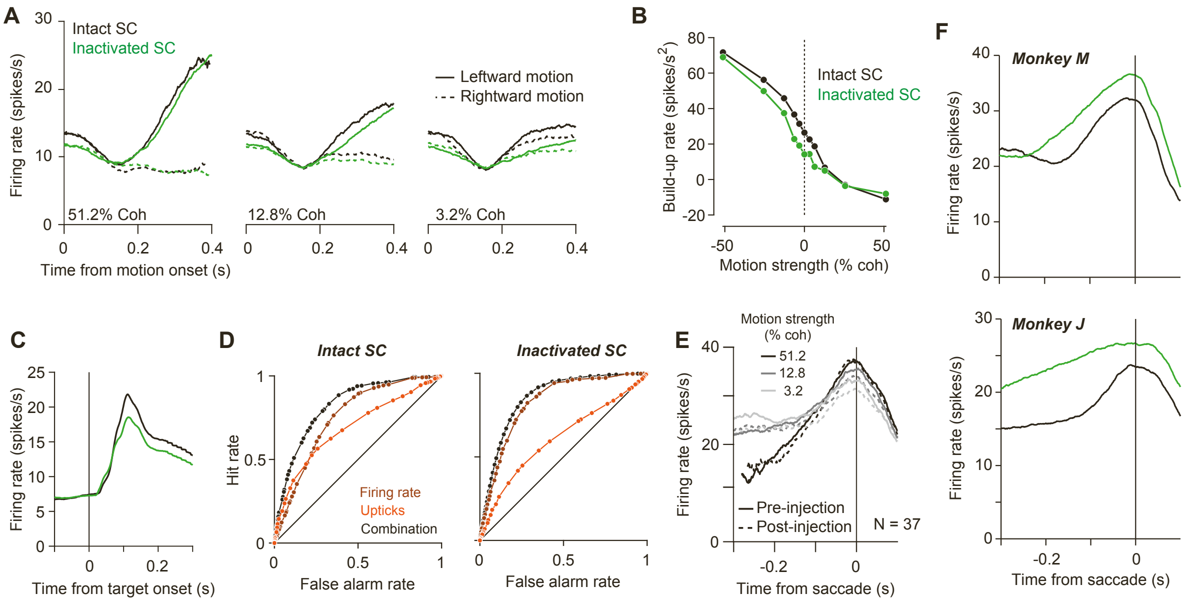

Figure S6: Additional effects of SC inactivation on LIP activity and saline control data, Related to Figure 6. A, Effect of SC inactivation on LIP activity early in the trial. Average of 58 LIP neurons before (black) and after (green) SC inactivation, aligned to motion onset. Activity is split by motion direction (line style) and plotted separately for a strong (*left*), intermediate (*middle*), and weak (*right*) motion strength. **B,** Build-up rate of LIP activity as a function of motion strength. Negative motion strengths represent leftward motion (i.e., supporting T_{in}). Build-up rates are calculated using the epoch 200–400 ms after motion onset. SC inactivation caused a small decrease in build-up rates. **C,** Effect of SC inactivation on the transient visual response to the targets. Average LIP activity before (black) and after (green) SC inactivation is aligned to the onset of the visual targets, which occurs at least 250 ms before the RDM stimulus is presented. **D,** ROC curves associated with the analysis in Fig. 6C. **E,** Same as Fig. 6A but for saline/sham sessions. Saline injection had a negligible effect on LIP activity at the end of the decision. **F,** Effect of SC inactivation on LIP activity aligned to saccade onset for Monkey M (top) and Monkey J (bottom).

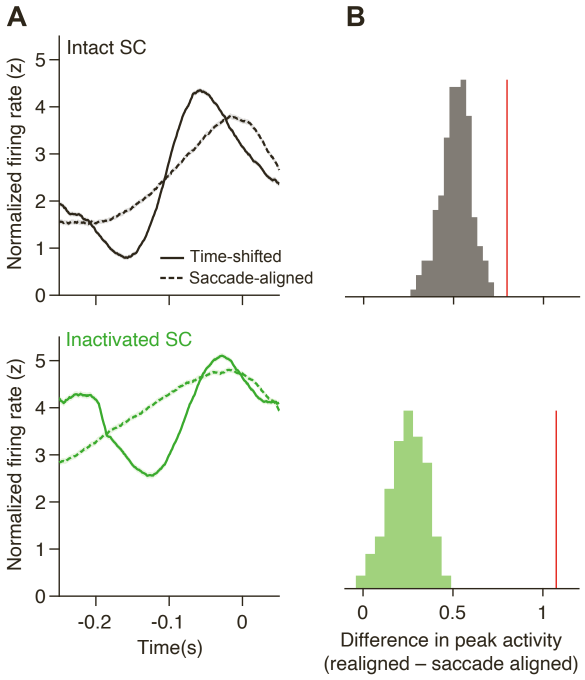

Figure S7: Realignment of upticks in LIP activity is not an artifact of the time-shift algorithm, Related to Figure 6. A, Application of the time-shift algorithm to simulated pre- and post-inactivation data sets (*top* and *bottom*, respectively). By design, the simulated data do not contain temporal misalignment. They are simulated as random Gaussian processes, matching the number of trials, the mean activity over time, and the autocovariance of the real data (see Methods). Dashed curves depict trial-averaged simulated data aligned to the saccade. Solid curves depict trial-averaged activity after time-shifting. Unsurprisingly, the algorithm realigns the simulated data such that the change in firing rate over time is greater after time-shifting. Note, however, that the realigned activity does not resemble the burst-aligned uptick shown in Fig 4A. **B,** Expected change in peak firing rate after time-shifting under the null hypothesis of no temporal misalignment. Distributions depict the change in peak trial-averaged activity after time-shifting for 200 simulated pre-inactivation data sets (*top*) and 200 post-inactivation data sets (*bottom*). Vertical red lines depict the same quantity computed from the real data. For both pre- and post-inactivation, the increase in peak firing rate after realigning the real data was significantly greater than that expected under the null hypothesis ($p < .005$).
